# Gastrointestinal Stromal Tumors, Somatic Mutations and Candidate Genetic Risk Variants

**DOI:** 10.1371/journal.pone.0062119

**Published:** 2013-04-18

**Authors:** Katie M. O'Brien, Irene Orlow, Cristina R. Antonescu, Karla Ballman, Linda McCall, Ronald DeMatteo, Lawrence S. Engel

**Affiliations:** 1 Department of Epidemiology, Gillings School of Global Public Health, University of North Carolina at Chapel Hill, Chapel Hill, North Carolina, United States of America; 2 Department of Epidemiology and Biostatistics, Memorial Sloan-Kettering Cancer Center, New York, New York, United States of America; 3 Department of Pathology, Memorial Sloan-Kettering Cancer Center, New York, New York, United States of America; 4 Department of Health Sciences Research, Mayo Clinic, Rochester, Minnesota, United States of America; 5 American College of Surgeons Oncology Group, Durham, North Carolina, United States of America; 6 Department of Surgery, Memorial Sloan-Kettering Cancer Center, New York, New York, United States of America; Centro di Riferimento Oncologico, IRCCS National Cancer Institute, Italy

## Abstract

Gastrointestinal stromal tumors (GISTs) are rare but treatable soft tissue sarcomas. Nearly all GISTs have somatic mutations in either the *KIT* or *PDGFRA* gene, but there are no known inherited genetic risk factors. We assessed the relationship between *KIT*/*PDGFRA* mutations and select deletions or single nucleotide polymorphisms (SNPs) in 279 participants from a clinical trial of adjuvant imatinib mesylate. Given previous evidence that certain susceptibility loci and carcinogens are associated with characteristic mutations, or “signatures” in other cancers, we hypothesized that the characteristic somatic mutations in the *KIT* and *PDGFRA* genes in GIST tumors may similarly be mutational signatures that are causally linked to specific mutagens or susceptibility loci. As previous epidemiologic studies suggest environmental risk factors such as dioxin and radiation exposure may be linked to sarcomas, we chose 208 variants in 39 candidate genes related to DNA repair and dioxin metabolism or response. We calculated adjusted odds ratios (ORs) and 95% confidence intervals (CIs) for the association between each variant and 7 categories of tumor mutation using logistic regression. We also evaluated gene-level effects using the sequence kernel association test (SKAT). Although none of the association p-values were statistically significant after adjustment for multiple comparisons, SNPs in *CYP1B1* were strongly associated with *KIT* exon 11 codon 557-8 deletions (OR = 1.9, 95% CI: 1.3-2.9 for rs2855658 and OR = 1.8, 95% CI: 1.2-2.7 for rs1056836) and wild type GISTs (OR = 2.7, 95% CI: 1.5-4.8 for rs1800440 and OR = 0.5, 95% CI: 0.3-0.9 for rs1056836). *CYP1B1* was also associated with these mutations categories in the SKAT analysis (p = 0.002 and p = 0.003, respectively). Other potential risk variants included *GSTM1, RAD23B* and *ERCC2*. This preliminary analysis of inherited genetic risk factors for GIST offers some clues about the disease's genetic origins and provides a starting point for future candidate gene or gene-environment research.

## Introduction

Gastrointestinal stromal tumors (GISTs) are soft tissue sarcomas that develop primarily in the stomach (60–70%) and small intestines (20–30%), but also appear in the rectum, colon, esophagus or omentum [1], [2]. These tumors are quite rare, with an estimated annual incidence of 6.8 cases per million individuals in the US between 1992 and 2000 [3], and 3300 to 6000 new US cases predicted each year [4], though systematic under-ascertainment of GIST cases implies the true rate is slightly higher [3], [5], [6]. Data from the National Cancer Institute's Surveillance, Epidemiology and End Results (SEER) program suggest that GISTs are more common in African-Americans than Caucasians (8.9 versus 4.5 cases per 1 million individuals per year, 1992-2002) but equally common in men and women [5]. Median age at diagnosis in the SEER population is 63 years.

Unlike other gastrointestinal neoplasms, more than 90% of GISTs express the *KIT* proto-oncogene, as measured by immunohistochemical analysis of CD117, the stem cell factor receptor protein encoded by *KIT* [7], [8]. In approximately 75% of GISTs, this CD117 overexpression is attributable to a gain-in-function mutation in the tyrosine kinase domain of KIT. Once mutated, *KIT* may encode tyrosine kinase receptors in which the tyrosine kinase domain can be activated in the absence of stem cell factor signaling, thereby stimulating excess, unregulated proliferation of the host tumor cells [8]–[10]. Another 10-15% of GISTs have mutations in the *PDGFRA* gene, another tyrosine kinase receptor encoding gene [8], [11].

Primary GIST-related *KIT* and *PDGFRA* mutations have been well characterized. Results from 3 population-based studies in Switzerland [12], Norway [13] and France [14] suggest that 50-60% of all GISTs have mutations in *KIT* exon 11, 5–10% in *KIT* exon 9, 3% in *KIT* exon 13, 1% in *KIT* exon 17, 2–5% in *PDGFRA* exon 12 and 2–6% in *PDGFRA* exon 18. The proportions observed in hospital-based or convenience samples are generally consistent with these estimates, with some variability due to inclusion criteria and small sample sizes [15]–[18]. 

Most GISTs with primary *KIT* or *PDGFRA* mutations respond to treatment with imatinib mesylate (STI571, Gleevec™, Novartis Pharmaceuticals, Basel, Switzerland), an inhibitor of the KIT and PDGFRA tyrosine kinase. Imatinib is more effective in patients with mutations in *KIT* exon 11 than in patients with no tumor mutations (wild type) or exon 9 mutations [19], [20]. Unfortunately, roughly half of the patients who initially respond to imatinib develop drug-resistant disease after prolonged treatment. This acquired resistance may be attributable to the development of secondary *KIT* mutations in residual tumor tissue [21]–[23].

While some *KIT* and *PDGFRA* germline mutations have been identified among families with multiple GIST cases [24], [25] and a few studies have identified single nucleotide polymorphisms (SNPs) associated with soft tissue sarcoma incidence (*MDM2* [26]), survival (*AhR* [27]), or specific translocations common in some types of soft tissue sarcoma (*XPG/ERCC5* [28]), no studies have looked for inherited genetic risk factors for sporadic GISTs. Though such studies are necessary to advance our understanding of disease etiology, recruitment of cases and compatible controls is limited by the disease's rarity. A population-based study with rapid case ascertainment and collection of detailed information on non-genetic risk factors would be especially arduous, as GISTs are often misclassified in reports to cancer surveillance systems [3], [5], [6].

Given these constraints, we decided to investigate the role of inherited genetic polymorphisms in GIST development by conducting a case-only analysis of the association between tumor mutation type (mutations in *KIT* exon 11, *KIT* exon 9, *PDGFRA*, or wild type) and 225 variants in 39 candidate genes using tumor and blood samples collected during a phase III clinical trial of adjuvant imatinib [29]. In previous studies, certain susceptibility loci have been linked to characteristic tumor mutations, or mutational “signatures”. These include associations between *GSTM1*-null genotype and *TP53* transversion mutations among bladder cancer patients [30], and certain functional polymorphisms in *XPD* and G:C→T:A *TP53* mutations among lung cancer patients [31]. Similarly, we hypothesized that the characteristic somatic mutations in the *KIT* and *PDGFRA* genes in GIST tumors may be mutational signatures that are causally linked to specific mutagens or susceptibility loci. To address this hypothesis, we selected candidate genes previously linked to soft tissue sarcoma or to environmental risk factors for soft tissue sarcoma. We included genes related to dioxin, phenoxyherbicide, insecticide, vinyl chloride, and radiation response, as well as variants in the previously identified *AhR, MDM2*, and *ERCC5* genes [32]–[38]. We also looked at polymorphisms in genes encoding proteins on the *AhR/ARNT* dioxin-response pathway (*CYP1A2*, *CYP1B1*, *HIF1A*, *NQO1*, and *G6PC/G6PT*) [39]–[41], other related metabolizing pathways (*ADH1A*, *ADH1B*, *ADH1C*, *ALDH18A1*, *ALDH1A1*, *ALDH1A2*, *ALDH1A3*, *ALDH1B1*, *ALDH1L1*, *ALDH1L2*, *ALDH2*, *CYP2B6*, *CYP2C8*, *CYP2C9*, *CYP2D6*, *CYP2E1*, *CYP3A4*, *GSTM1*, *GSTT1*, *GSTP1*, *HNF4A*, *NAT2*, *NFE2L2, NOS2A*, *PTGS2/COX2*, and *SULT1A1*) [42]–[45] and *TP53*, a tumor suppressor and cell cycle regulation gene closely related to *MDM2* [26]. Additionally, we selected several DNA repair genes (*ERCC2*, *RAD23B*, *XPA,* and *XPC*) in the same DNA repair pathway as *ERCC5*, as polymorphisms in these nucleotide excision repair genes can affect individual sensitivity to carcinogen-induced DNA damage [46]. As our main objective was to conduct a preliminary assessment of these candidate genes rather than any specific variants, we conducted gene-level as well as SNP-level association analyses.

## Materials and Methods

### Study population

In total, 713 individuals participated in American College of Surgeons Oncology Group (ACOSOG) Z9001, a multicenter, phase III, randomized, double-blind study of adjuvant imatinib (Gleevec™) versus placebo for patients with resected, primary GISTs conducted between July 1, 2002 and April 18, 2007. Cases were eligible if they had a localized tumor of at least 3 cm that tested positive for CD117 by immunohistochemical analysis with the Dako antibody (DakoCytomation, Copenhagen, Denmark). Additional information on the Z9001 trial is published elsewhere [29].

This genetic ancillary study includes the first 333 Z9001 participants who provided a blood sample and consented in writing to unspecified future research using their blood and tumor tissue samples. After removing individuals missing mutation data (n = 52) or more than 10% of their genotype data (n = 2), 279 participants remained. Information on each participant's race, age, sex, and tumor size, site, stage, grade and mitotic rate was available from the parent study. The study protocol was approved by the Institutional Review Boards of Memorial Sloan-Kettering Cancer Center and the University of North Carolina. All participants provided written informed consent.

### Variant selection

Once we selected our candidate genes, we identified single nucleotide polymorphisms (SNPs) or deletions within those genes that potentially affected function and had minor allele frequencies (MAF) equal to or greater than 10% in the HapMap CEU population [47]. This included nonsense, missense and splice site mutations, as well as mutations in seed microRNA regions or transcription binding sites. All selected nonsense, missense or splice site mutations were in or near coding regions (within 2000 and 500 base pairs of the 5′ and 3′ ends of the region, respectively). SNPs that did not pass the design phase (designability score <1 or final score <0.7) were replaced with surrogate SNPs in high linkage disequilibrium with the original candidate SNP.

### Laboratory Analysis

During Z9001 enrollment, all tumor and blood specimens were banked with the ACOSOG Central Specimen Bank at Washington University School of Medicine in St. Louis, Missouri, then DNA extracted from these blood samples was sent to Memorial Sloan-Kettering Cancer Center (MSKCC) for storage at −80°C until analysis. Each sample was genotyped using the GoldenGate genotyping assay (Illumina Inc., San Diego, CA) [48], which consisted of allele-specific extension/ligation methodology followed by universal primer polymerase chain reaction (PCR) amplification regions for the candidate SNPs. Allele-specific oligos and locus-specific oligos hybridized directly to the genomic DNA, upstream and downstream from the targeted SNP before the universal PCR reaction took place [49]. For internal quality control purposes, twenty-seven participants underwent duplicate genotype analysis. Concordance for duplicate samples was 99.9%. SNPs were excluded if they were mono-allelic (n = 3), had a MAF less than 5% in our study samples (n = 6), showed poor clustering (n = 7), or had no individuals homozygous for the minor allele at some levels of the outcome (n = 1), leaving 208 SNPs in the final analysis.

Deletions in GSTM1 and GSTT1 were detected using multiplex PCR utilizing sets of target specific and housekeeping gene specific primers [50]. Here, individuals with no copies of the polymorphism of interest (null genotype) were differentiated from those who had one or two copies (wild type).

DNA for mutation analysis was extracted from tumor tissue that was snap-frozen and then analyzed as previously described [15], [51]. Briefly, all cases were first tested for *KIT* exon 11 mutations via PCR analysis using Platinum TaqDNA Polymerase High Fidelity (Life Technologies, Inc., Gaithersburg, MD). Tumors without exon 11 mutations were then subjected to PCR analysis using primers for *KIT* exon 9, 13, 14 and 17 and *PDGFRA* exon 12 and 18.

### Statistical Analysis

Participants were categorized dichotomously based on the presence or absence of a specific mutation type. The following outcomes were considered: i) a deletion of *KIT* exon 11 codons 557–558, ii) any other (i.e. non-codon 557-8) *KIT* exon 11 deletion, iii) a *KIT* exon 11 insertion, iv) A *KIT* exon 11 point mutation, v) a *KIT* exon 9, exon 13, exon 14, or exon 17 mutation, vi) a *PDGFRA* exon 18 or 12 mutation, and vii) no *KIT* or *PDGFRA* mutation (wild type). Although differentiation by non-exon 11 *KIT* mutations would have been preferable, the prevalence of exon 9, 13, 14 and 17 mutations was too low for independent outcome assessment.

We conducted descriptive analyses of selected demographic variables and tumor characteristics, both overall and stratified by gender and race (white vs. non-white). We also compared the covariate distributions of our study population with the remaining Z9001 trial participants to look for possible indications of bias. For each variant, we calculated the race-specific MAF and Pearson χ^2^ p-value for the association between genotype and race. We used Fisher's exact test when one or more cells had less than 5 observations. Additionally, we conducted a crude case-control analysis by comparing the genotype distributions among the white participants (n = 273) to the genotype distributions among individuals of European descent using the HapMap database [47]. Individuals with missing mutation data were included in these descriptive analyses.

The association between germline polymorphisms and somatic mutations was analyzed using logistic regression. We obtained odds ratios (ORs), 95% confidence intervals (CIs) and p-values for each SNP-mutation combination, adjusting for race, sex, and age at diagnosis. We coded genotypes as ordinal variables (0 = homozygous for the major allele, 1 = heterozygous, 2 = homozygous for the minor allele) and estimated per-allele ORs and 1 df trend tests. All p-values were corrected for multiple testing by controlling for the false discovery rate [52].

Gene-level association tests were conducted using the sequence kernel association test (SKAT) developed by Wu et al [53], [54]. Here, SNPs are grouped based on prior biological knowledge, in this case occurrence in the same gene, and analyzed using a logistic kernel-machine-based multi-locus test. This method requires fewer hypothesis tests than standard techniques and improves power to detect the effect of an untyped, causal locus by incorporating data from several correlated surrogate SNPs. This method also allows for covariate adjustment, nonlinear effects, and epistasis.

Briefly, this method uses a modified version of the variance component score test to assess whether the variance of subject-specific random effects differs from 0. The subject-specific model for each of *n* individuals takes the form: 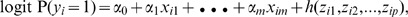



where y_i_ is the outcome for individual *i*, *x*_i1_ to *x*_im_ are the covariate values for individual i, α_0_ to α_m_ are the regression parameters, *z*_i1_ to *z*_ip_ are the genotypes for individual *i* at genotypes 1 to *p*, and *h*_i_ = *h*(*z*_i1_, *z*_12_,…*z*_ip_) = *h*(***Z*** **_i_**) = 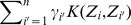 is a function for the subject-specific random effect defined by a positive, definite kernel function of the form K(•,•) and some γ_i_, …, γ_n_. Assuming **h** follows an arbitrary distribution with a mean of 0 and variance τ**K**, testing the null hypothesis H_0_: h(**Z**) = 0 is equivalent to testing H_0_: τ = 0, which can be accomplished using a variance-component score statistic [55]:
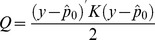



where logit 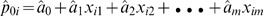. To obtain a p-value, we can compare *Q* to a scaled χ^2^ distribution with scale parameter κ and degrees of freedom ν, which are modified to account for correlation between SNPs in the same SNP-set (for further explanation, see Appendix A in Wu et al [54]). In this analysis, we opted to use a kernel that models identity-by-state (IBS), or the number of alleles shared by a pair of individuals. This kernel is the most powerful option when epistatic effects may be present.

## Results

Descriptive analyses are shown in Table 1. The median age for included participants was 58.0 years (range 18–85). Approximately half of the population was male (51%) and the majority were white (82%). Most tumors were located in the stomach (66%) or small intestines (31%) and were between 5 and 10 cm in diameter.

**Table 1:** Demographic information and tumor characteristics of patients included in genotyping ancillary study.

	Overall Sample	Sex Stratified		Race Stratified	
	N = 279	Male (n = 142)	Female (n = 137)	White (n = 229)	Other (n = 50)
**Age: Median (range)**	58.0 (18–85)	57.0 (18–85)	58.0 (18–81)	59.0 (18–85)	53.0 (27–78)
**Sex: N (%)**					
Male	142 (51)	---	---	---	---
Female	137 (49)	---	---	---	---
**Race: N (%)**					
White	229 (82)	122 (86)	107 (78)	---	---
Other	50 (18)	20 (14)	30 (22)	---	---
**Tumor Size: Median (range)**	6.5 (3.0–37.0)	6.0 (3.0–37.0)	6.5 (3.0–28.0)	6.5 (3.0–37.0)	6.0 (3.1–30.0)
**Tumor Size: N(%)**					
<5 cm	79 (28)	41 (29)	38 (28)	65 (28)	14 (28)
5-10 cm	146 (52)	72 (51)	74 (54)	119 (52)	27 (54)
>10 cm	54 (19)	29 (20)	25 (18)	45 (20)	9 (18)
**Mitotic Rate: Median (range)**	3 (0–351)	3 (0–351)	3 (0–207)	3 (0–351)	4.5 (0–81)
**Mitotic Rate: N(%)**					
<5	156 (60)	77 (58)	79 (63)	132 (62)	24 (50)
≥5	104 (40)	57 (42)	47 (37)	80 (38)	24 (50)
Missing	19	8	11	17	2
**Tumor Location: N(%)**					
Stomach	182 (66)	97 (69)	85 (63)	146 (64)	36 (74)
Small Intestine	85 (31)	39 (28)	46 (34)	77 (34)	8 (16)
Rectum	2 (1)	1 (1)	1 (1)	1 (0)	1 (2)
Other	8 (3)	4 (3)	4 (3)	4 (2)	4 (8)
Missing	2	1	1	1	1
**Mutation Type: N(%)**					
Exon 9	15 (5)	9 (6)	6 (4)	15 (7)	0 (0)
Exon 11	195 (70)	95 (67)	100 (73)	153 (67)	42 (84)
Exon 13	3 (1)	0 (0)	3 (2)	2 (1)	1 (2)
Exon 14	1 (0)	1 (1)	0 (0)	1 (0)	0 (0)
Exon 17	0 (0)	0 (0)	0 (0)	0 (0)	0 (0)
PDGFRA	29 (10)	21 (15)	8 (6)	25 (11)	4 (8)
Wild type	36 (13)	16 (11)	20 (15)	33 (14)	3 (6)
**Exon 11 mutation type: N(%)**					
557-8 deletion	66 (34)	33 (35)	33 (33)	51 (33)	15 (36)
Other deletion	45 (23)	25 (26)	20 (20)	34 (22)	11 (26)
Insertion	28 (14)	14 (15)	14 (14)	23 (15)	5 (12)
Point Mutation	56 (29)	23 (24)	33 (33)	45 (29)	11 (26)
**PDGFRA mutation type: N(%)**					
D842V	12 (41)	10 (48)	2 (25)	10 (40)	2 (50)
Other	17 (59)	11 (52)	6 (75)	15 (60)	2 (50)

70% of evaluated tumors had exon 11 *KIT* mutations, 10% had *PDGFRA* mutations and 13% had no identified *KIT* or *PDGFRA* mutations. Non-white participants were younger, on average (53.0 years vs. 59.0 years), and more likely to have stomach tumors (74% vs. 64%) and exon 11 *KIT* mutations (84% vs. 67%). The most common exon 11 *KIT* mutation was a deletion at codons 557–558 (34%).

Compared with other ACOSOG Z9001 participants, the individuals included in this genotyping substudy have similar demographic and tumor characteristics (Table 2). A somewhat higher proportion of participants in this ancillary study were white (82% versus 76%), but our subpopulation had nearly identical age, gender, tumor size, mitotic rate, tumor location, and tumor mutation type distributions to the full patient pool.

**Table 2:** Comparison of patients included in the genetic ancillary study to the remainder of the Z9001 clinical trial patients.

	Ancillary study (n = 279)	Remaining Z9001 patients (n = 436)
**Age: Median (range)**	58.0 (18–85)	59.0 (21–91)
**Sex: N (%)**		
Male	142 (51)	219 (50)
Female	137 (49)	217 (50)
**Race: N (%)**		
White	229 (82)	332 (76)
Other	50 (18)	104 (24)
**Tumor Size: Median (range)**	6.5 (3.0–37.0)	6.6 (3.0–43.0)
**Tumor Size: N(%)**		
<5 cm	79 (28)	118 (27)
5–10 cm	146 (52)	112 (49)
>10 cm	54 (19)	105 (24)
**Mitotic Rate: Median (range)**	3 (0–351)	3 (0–289)
**Mitotic Rate: N(%)**		
<5	156 (60)	235 (65)
≥5	104 (40)	126 (35)
Missing	19	75
**Tumor Location: N(%)**		
Stomach	182 (66)	263 (61)
Small Intestine	85 (31)	142 (33)
Rectum	2 (1)	8 (2)
Other	8 (3)	22 (5)
Missing	1	2
**Mutation Type: N(%)**		
Exon 9	15 (5)	20 (9)
Exon 11	195 (70)	148 (64)
Exon 13	3 (1)	6 (3)
Exon 14	1 (0)	0 (0)
Exon 17	0 (0)	1 (0)
PDGFRA	29 (10)	27 (12)
Wild type	36 (13)	28 (12)
Missing	0	206
**Exon 11 mutation type: N(%)**		
557-8 deletion	66 (34)	41 (28)
Other deletion	45 (23)	34 (23)
Insertion	28 (14)	18 (12)
Point Mutation	56 (29)	55 (37)
**PDGFRA mutation type: N(%)**		
D842V	12 (41)	15 (56)
Other	17 (59)	12 (44)

Genotype distributions of the 208 variants varied substantially by race (Table S1), but genotype frequencies among whites in our study population were very similar to the HapMap CEU sample for the 204 SNPs available in both populations. Notable discrepancies included SNPs on several aldehyde dehydrogenase genes, *ALDH1A3*, *ALDH1A2*, *ALDH1L1* and *ALDH1L2*, and two DNA repair genes, *ERCC2* and *XPC*.

**Figure 1:**
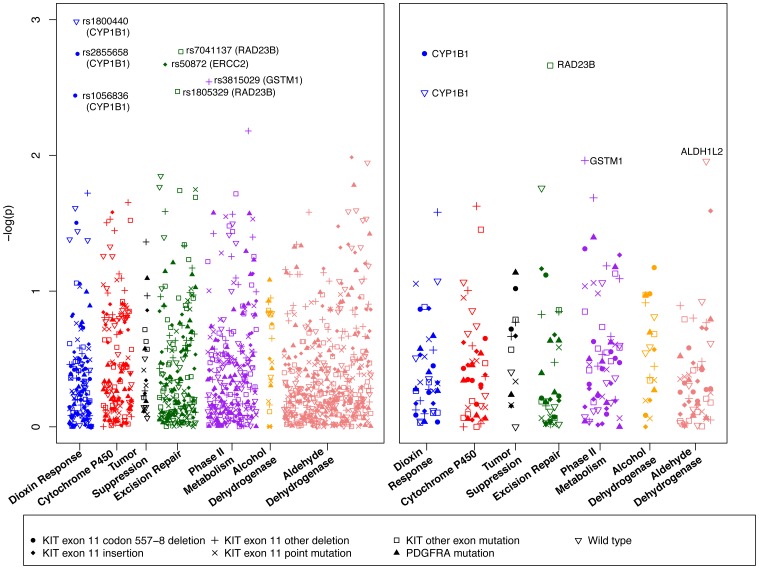
Log p-values for individual variant (left) and SKAT (right) analyses by functional group and tumor mutation type.

The associations between each genetic variant and possible outcome are depicted in Figure 1, with the strength of the association quantified by the inverse of the log of the p-value. While no SNPs were statistically significant after controlling for an FDR level of 25%, some interesting patterns emerged. Most notably, minor alleles at *CYP1B1* rs1056836 and rs2855658 were positively associated with a deletion at *KIT* exon 11 codons 557-8 (OR = 1.81, 95% CI: 1.21–2.71 and OR = 1.91, 95% CI: 1.27–2.86, respectively), while variation in another *CYP1B1* SNP, rs1800440, was positively associated with wild type tumors (OR = 2.65, 95% CI: 1.48–4.76). Having a rare variant at rs1056836 was inversely associated with wild type tumors (OR = 0.54, 95% CI: 0.32–0.92).

Minor alleles in two *RAD23B* SNPs, rs7041137 and rs1805329, were more common among tumors with *KIT* exon 9, 13, or 14 mutations (OR_rs7041136_ = 3.05, 95% CI: 1.52–6.12 and OR_rs1805329_ = 3.24, 95% CI: 1.48–7.11) than tumors without such mutations. The rare form of a third *RAD23B* SNP, rs1805334, was also positively associated with non- exon 11 *KIT* mutations (OR = 2.45, 95% CI: 1.16–5.14).

rs50872 in *ERCC2* was the strongest risk factor for *KIT* exon 11 insertion mutations (OR = 2.68, 95% CI: 1.43–5.04) and the rare variant of rs3815029 in *GSTM1* was inversely associated with non-codon 557-8 *KIT* exon 11 deletions (OR = 0.43, 95% CI: 0.25,0.75). Based on the above evidence that at least one variant in *CYP1B1*, *RAD23B*, *ERCC2*, or *GSTM1* was associated with one or more GIST mutation types at p<0.005, we provided a detailed evaluation of the estimated effects for all of the variants in these four key genes (Table 3). Effect estimates and p-values for the remaining variants were included in Table S2. This table includes results for rs4646755 in *ALDH1L1* and rs3731149 in *XPC*, the strongest risk factors for *PDGFRA* mutations and *KIT* exon 11 point mutations, respectively, both of which had p-values of 0.02.

**Table 3:** Minor allele frequencies (MAF), Odds Ratios (ORs) and association p-values for SNPs in CYP1B1, ERCC2, GSTM1, and RAD23B by mutation type.

Gene	SNP/ variant		KIT exon 11 codon 557-8 deletion	KIT exon 11 insertion	KIT exon 11 other deletion	KIT exon 11 point mutation	Other KIT mutation	PDGFRA mutation	Wild type
CYP1B1	rs1056836	MAF^a^	0.41/0.57	0.54/0.53	0.50/0.54	0.57/0.52	0.64/0.53	0.53/0.53	0.68/0.51
		OR (95% CI)	1.81 (1.21, 2.71)	1.01 (0.58, 1.75)	1.11 (0.71, 1.73)	0.81 (0.53, 1.22)	0.71 (0.35, 1.42)	0.93 (0.54, 1.61)	0.54 (0.32, 0.92)
		p-value	0.004	1.0	0.6	0.3	0.3	0.8	0.02
CYP1B1	rs1800440	MAF^a^	0.11/0.20	0.16/0.18	0.14/0.19	0.13/0.19	0.31/0.17	0.24/0.17	0.32/0.16
		OR (95% CI)	0.52 (0.28, 0.94)	0.79 (0.37, 1.67)	0.78 (0.42, 1.46)	0.66 (0.36, 1.20)	1.88 (0.91, 3.86)	0.75 (0.39, 1.42)	2.65 (1.48, 4.76)
		p-value	0.03	0.5	0.4	0.2	0.1	0.4	0.001
CYP1B1	rs2855658	MAF^a^	0.40/0.58	0.55/0.53	0.50/0.54	0.58/0.52	0.64/0.53	0.53/0.53	0.67/0.51
		OR (95% CI)	1.91 (1.27, 2.86)	0.94 (0.54, 1.63)	1.12 (0.71, 1.75)	0.77 (0.51, 1.17)	0.71 (0.35, 1.42)	0.93 (0.54, 1.61)	0.56 (0.32, 0.96)
		p-value	0.002	0.8	0.6	0.2	0.3	0.8	0.04
ERCC2	rs13181	MAF^a^	0.33/0.33	0.41/0.32	0.36/0.32	0.32/0.33	0.39/0.32	0.33/0.33	0.22/0.34
		OR (95% CI)	1.03 (0.68, 1.55)	1.53 (0.87, 2.68)	1.24 (0.78, 1.98)	0.96 (0.62, 1.50)	1.20 (0.60, 2.38)	0.96 (0.54, 1.69)	0.47 (0.26, 0.88)
		p-value	0.9	0.1	0.4	0.9	0.6	0.9	0.02
ERCC2	rs171140	MAF^a^	0.44/0.43	0.39/0.43	0.33/0.45	0.42/0.43	0.44/0.43	0.38/0.44	0.60/0.41
		OR (95% CI)	1.16 (0.77, 1.76)	0.81 (0.45, 1.45)	0.67 (0.41, 1.10)	0.94 (0.61, 1.45)	0.88 (0.44, 1.76)	1.33 (0.74, 2.39)	1.98 (1.15, 3.42)
		p-value	0.5	0.5	0.1	0.8	0.7	0.3	0.01
ERCC2	rs1799787	MAF^a^	0.23/0.25	0.30/0.24	0.26/0.25	0.24/0.25	0.33/0.24	0.26/0.25	0.19/0.26
		OR (95% CI)	0.95 (0.60, 1.51)	1.40 (0.76, 2.57)	1.15 (0.68, 1.93)	0.95 (0.58, 1.55)	1.36 (0.66, 2.79)	0.92 (0.49, 1.71)	0.58 (0.30, 1.10)
		p-value	0.8	0.3	0.6	0.8	0.4	0.8	0.1
ERCC2	rs3916874	MAF^a^	0.24/0.22	0.18/0.23	0.21/0.23	0.21/0.23	0.19/0.23	0.24/0.22	0.26/0.22
		OR (95% CI)	1.20 (0.76, 1.91)	0.70 (0.34, 1.46)	0.94 (0.54, 1.64)	0.95 (0.57, 1.59)	0.77 (0.33, 1.80)	0.97 (0.51, 1.85)	1.28 (0.71, 2.28)
		p-value	0.4	0.3	0.8	0.8	0.5	0.9	0.4
ERCC2	rs50871	MAF^a^	0.36/0.45	0.45/0.42	0.33/0.44	0.43/0.43	0.42/0.43	0.53/0.41	0.57/0.41
		OR (95% CI)	0.74 (0.49, 1.11)	1.08 (0.62, 1.89)	0.69 (0.43, 1.11)	1.04 (0.68, 1.58)	0.80 (0.41, 1.57)	0.64 (0.37, 1.13)	1.73 (1.03, 2.93)
		p-value	0.1	0.8	0.1	0.9	0.5	0.1	0.04
ERCC2	rs50872	MAF^a^	0.24/0.21	0.39/0.20	0.13/0.24	0.21/0.22	0.33/0.21	0.14/0.23	0.18/0.23
		OR (95% CI)	1.21 (0.75, 1.95)	2.68 (1.43, 5.04)	0.47 (0.24, 0.91)	0.88 (0.52, 1.49)	1.91 (0.90, 4.04)	2.09 (0.94, 4.66)	0.79 (0.41, 1.54)
		p-value	0.4	0.002	0.03	0.6	0.1	0.1	0.5
GSTM1	deletion	MAF^a^	0.38/0.55	0.50/0.51	0.47/0.52	0.56/0.50	0.76/0.49	0.59/0.50	0.56/0.50
		OR (95% CI)	1.91 (1.07, 3.39)	1.07 (0.48, 2.36)	1.17 (0.61, 2.25)	0.75 (0.41, 1.38)	0.33 (0.10, 1.04)	1.38 (0.62, 3.04)	0.89 (0.43, 1.86)
		p-value	0.03	0.9	0.6	0.4	0.1	0.4	0.8
GSTM1	rs3815029	MAF^a^	0.42/0.37	0.34/0.39	0.24/0.41	0.45/0.37	0.39/0.38	0.40/0.38	0.40/0.38
		OR (95% CI)	1.32 (0.87, 2.00)	0.76 (0.40, 1.41)	0.43 (0.25, 0.75)	1.46 (0.93, 2.28)	0.98 (0.48, 2.04)	1.00 (0.56, 1.81)	1.16 (0.68, 1.99)
		p-value	0.2	0.4	0.003	0.1	1.0	1.0	0.6
RAD23B	rs10868	MAF^a^	0.10/0.10	0.09/0.10	0.09/0.10	0.07/0.11	0.14/0.10	0.12/0.10	0.14/0.10
		OR (95% CI)	1.02 (0.51, 2.04)	0.79 (0.28, 2.19)	0.90 (0.39, 2.05)	0.58 (0.26, 1.33)	1.34 (0.46, 3.86)	0.82 (0.33, 2.02)	1.49 (0.66, 3.37)
		p-value	1.0	0.6	0.8	0.2	0.6	0.7	0.3
RAD23B	rs1805329	MAF^a^	0.16/0.18	0.13/0.18	0.16/0.18	0.17/0.17	0.39/0.16	0.16/0.17	0.17/0.17
		OR (95% CI)	0.95 (0.55, 1.64)	0.69 (0.29, 1.61)	0.97 (0.51, 1.86)	1.03 (0.58, 1.82)	3.24 (1.48, 7.11)	1.19 (0.54, 2.61)	0.77 (0.38, 1.55)
		p-value	0.8	0.4	0.9	0.9	0.003	0.7	0.5
RAD23B	rs1805330	MAF^a^	0.06/0.09	0.07/0.08	0.11/0.08	0.08/0.08	0.14/0.08	0.07/0.08	0.08/0.08
		OR (95% CI)	0.65 (0.30, 1.38)	0.80 (0.29, 2.20)	1.29 (0.64, 2.61)	0.94 (0.45, 1.94)	2.22 (0.83, 5.95)	1.26 (0.45, 3.51)	1.36 (0.55, 3.36)
		p-value	0.3	0.7	0.5	0.9	0.1	0.7	0.5
RAD23B	rs1805334	MAF^a^	0.23/0.23	0.18/0.23	0.24/0.22	0.22/0.23	0.42/0.21	0.16/0.23	0.21/0.23
		OR (95% CI)	1.10 (0.68, 1.79)	0.75 (0.36, 1.56)	1.23 (0.70, 2.16)	1.01 (0.60, 1.69)	2.45 (1.16, 5.14)	1.72 (0.79, 3.75)	0.74 (0.39, 1.41)
		p-value	0.7	0.4	0.5	1.0	0.02	0.2	0.4
RAD23B	rs7041137	MAF^a^	0.27/0.29	0.21/0.29	0.30/0.28	0.28/0.28	0.53/0.27	0.24/0.29	0.26/0.29
		OR (95% CI)	0.93 (0.61, 1.42)	0.70 (0.37, 1.34)	1.09 (0.68, 1.74)	0.98 (0.63, 1.54)	3.05 (1.52, 6.12)	1.27 (0.69, 2.34)	0.90 (0.52, 1.59)
		p-value	0.7	0.3	0.7	0.9	0.002	0.4	0.7
^a^MAF among those with mutation/MAF among those without mutation.									

These patterns were preserved in the gene-level SKAT analysis (Figure 1, Table 4, and Table S3), with *CYP1B1* again associated with *KIT* exon 11 codon 557-8 deletions and wild type tumors (p = 0.002 and 0.003, respectively); strong associations between *RAD23B* and KIT exon 9, 13 or 14 mutations (p = 0.002); and *GSTM1* and non-codon 557-8 *KIT* exon 11 deletions (p = 0.01). *ALDH1L2* was also strongly associated with wild type tumors (p = 0.01). As for the other three possible tumor subtypes, *ALDH2* was associated with *KIT* exon 11 insertions (p = 0.03) and the null *GSTT1* genotype was associated with *PDGFRA*-mutated tumors (p = 0.04). No genes were associated with *KIT* exon 11 point mutations (p<0.05).

**Table 4:** P-values for sequence kernel association test (SKAT) for CYP1B1, ERCC2, GSTM1, and RAD23B, by mutation type.

Gene	KIT exon 11 codon 557-8 deletion	KIT exon 11 insertion	KIT exon 11 other deletion	KIT exon 11 point mutation	Other KIT mutation	PDGFRA mutation	Wild type
CYP1B1	0.002	0.8	0.8	0.3	0.1	0.9	0.003
ERCC2	0.6	0.1	0.1	0.9	0.6	0.4	0.02
GSTM1	0.05	0.7	0.01	0.1	0.1	0.9	0.8
RAD23B	0.9	0.6	1.0	0.8	0.002	0.2	0.8

Although the effect estimates were very imprecise, the associations between the rare alleles of *CYP1B1* SNPs rs1056836 and rs2855658 and *KIT* exon 11 codon 557-8 deletions were even stronger when the analysis was limited to small intestinal tumors (OR_rs1056836_ = 5.18, 95% CI: 2.07, 12.95 and OR_rs2855658_ = 5.17, 95% CI: 2.05, 13.03). Neither SNP was associated with the outcome in stomach GISTs. No other clear patterns emerged in site-specific subanalyses (data not shown).

## Discussion

In this preliminary investigation of genetic risk factors for GIST tumor subtypes we identified several genes and SNPs worthy of further investigation. This included SNPs on two xenobiotic metabolizing genes, *CYP1B1* and *GSTM1*, and two DNA repair genes, *RAD23B* and *ERCC2*. Further exploration of the relationship between GISTs and aldehyde dehydrogenase genes or other DNA repair genes (e.g. *XPC*), may also be warranted.

*CYP1B1* encodes a cytochrome *P*450 enzyme that is involved with phase I metabolism of PAHs, dioxin, and other chemicals [43]. Two of the *CYP1B1* SNPs we assessed have previously been linked to cancer. This included the rare variant at rs1056836, a missense mutation, which has been linked to increased risk of lung cancer [56], [57], multiple myeloma [39] and head and neck cancer [58], [59], with a possible inverse association with pancreatic cancer [60]. Previous evaluations of the SNP's association with breast, colorectal, endometrial and prostate cancer have produced mostly null findings [61]–[65]. The rare allele of rs1800440, another missense mutation, was also associated with lung and head and neck cancer [56], [59], with no reported association with breast or colorectal cancer [62], [66]. However, this SNP did exhibit an inverse association with endometrial cancer [61], [65]. The remaining *CYP1B1* SNP, rs2855658, is located in a seed microRNA region, but has no previously established links to cancer.

Although there is little evidence of a link between cancer and the specific *RAD23B*, *ERCC2*, and *GSTM1* variants identified here, previous studies have observed associations between one or more types of cancer and other variants on these three genes. For example, SNPs in *RAD23B* have been linked to esophageal [67] and bladder [68] cancers and one SNP near *RAD23B* was strongly associated with breast cancer in a genome-wide association study [69]. *ERCC2* has also been linked to bladder cancer [68] and a large meta-analysis completed in 2006 reported statistically significant associations between *ERCC2* SNPs and skin, breast and lung cancer [70]. Neither *RAD23B* nor *ERCC2* have been linked to any type of sarcoma. Like the seed microRNA and missense mutation SNPs in *CYP1B1* that were strongly associated with tumor mutations in the present study, some of the identified *RAD23B* and *ERCC2* SNPs also have potentially functional roles. For example, rs13181 on *ERCC2* is a missense mutation, as is rs1805329 on *RAD23B*. Additionally, *RAD23B*'s rs1805330 is a splice site mutation and rs10868 and rs1805334 are located on transcription binding sites. As previously discussed, both *RAD23B* and *ERCC2* are nucleotide excision repair genes. Polymorphisms in these and other DNA repair genes could impair an individual's DNA damage response and affect their carcinogen sensitivity [46].

*GSTM1* is one of several genes encoding glutathione *S*-transferases, which are phase II xenobiotic metabolizing enzymes responsible for carcinogen activation or detoxification [45]. In previous studies, *GSTM1* deletions have been linked to osteosarcoma incidence [71] and recurrence [72], with a non-statistically significant positive association with soft tissue sarcoma mortality [73]. Other studies of *GSTM1* deletions have identified positive associations between the null genotype and a variety of cancers, included oral [45], colorectal [74], cervical [75], and bladder [76].

None of the association p-values were statistically significant after adjustment for multiple comparisons, whether we applied a false discovery rate correction of 25% or even 50%. While this implies that the observed associations may be due to chance, it should be noted that this was the first investigation of inherited risk factors for GISTs and our main study purpose was to identify variants worthy of further exploration. This study may also have limited generalizability. Study subjects were drawn from a predominantly white clinical trial population, and our findings may not be applicable to other racial groups or to all socioeconomic groups. As the HapMap CEU population is made up of 60 parent-child trios, it may not be an adequate comparison group for our population, especially since we were unable to adjust for unequal distributions of age, gender or other potential confounders.

Outcome misclassification is also a potential concern, as tumors with *KIT* exon 11 mutations were not assessed for other *KIT* or *PDGFRA* mutations and we did not test for *PDGFRA* exon 14 mutations in any tumors. However, previous reports suggest that GISTs with 2 or more mutations are rare (<5%) [12], [14], as are *PDGFRA* exon 14 mutations (<1%) [11], [77]. Thus, outcome misclassification is unlikely to be a substantial source of bias. While we have only limited evidence that our outcome classification system corresponds to distinct carcinogenic processes in GISTs, linking genetic polymorphisms to tumor phenotypes is valuable for generating etiologic hypotheses [37], [38].

In this small, yet novel, case-only study of genetic risk factors for GIST tumor subtypes we identified several variants in *CYP1B1*, *RAD23B*, *GSTM1*, and *ERCC2* that we believe are worthy of further investigation. We hope that this exploratory analysis serves as a starting point for future research on genetic and environmental causes of these rare and understudied tumors.

## Supporting Information

Table S1 **Minor allele frequencies (MAF) and p-values for comparison of genotype frequencies: Z9001 genotyped whites (n = 273) versus non-whites (n = 58) and genotyped Z9001 whites versus the HapMap CEU population (n = 180).**
(PDF)

Table S2 **Minor allele frequencies (MAF), Odds Ratios (ORs) and association p-values by mutation type.**
(PDF)

Table S3 **P-values for sequence kernel association test (SKAT) for remaining genes, by mutation type.**
(PDF)
