# Supplementary material for: Gastrointestinal Stromal Tumors, Somatic Mutations and Candidate Genetic Risk Variants
Source: PLoS One. 2013 Apr 18;8(4):e62119. doi: 10.1371/journal.pone.0062119 (PMC3630216; doi:10.1371/journal.pone.0062119)
Supplement: Table S1 — Minor allele frequencies (MAF) and p-values for comparison of genotype frequencies: Z9001 genotyped whites (n = 273) versus non-whites (n = 58) and genotyped Z9001 whites versus the HapMap CEU population (n = 180). (PDF) [file pone.0062119.s001.pdf]

Supplementary Table S1: Minor allele frequencies (MAF) and p-values for comparison of genotype frequencies: Z9001 genotyped whites (n=273) versus non-whites (n=58) and genotyped Z9001 whites versus the HapMap CEU population (n=180)

GENE	VARIANT	MAF, study whites	MAF, study non-whites	p-value whites vs. non-whites*	MAF, HapMap CEU	p-value whites vs. HapMap CEU*
ADH1A	rs12508502	0.09	0.05	0.37	0.09	0.69
ADH1B	rs1042026	0.28	0.30	0.03	0.28	0.96
ADH1B	rs17033	0.09	0.05	0.37	0.10	0.69
ADH1C	rs1693482	0.39	0.13	2.3E-07	0.48	0.10
AHR	rs2066853	0.12	0.38	1.2E-09	0.09	0.32
AHR	rs713150	0.24	0.22	0.83	0.22	0.76
ALDH18A1	rs10509688	0.16	0.16	0.12	0.21	0.09
ALDH18A1	rs1053905	0.45	0.16	4.1E-15	0.48	0.55
ALDH18A1	rs11188388	0.08	0.09	0.54	0.06	0.81
ALDH18A1	rs11188394	0.35	0.05	3.6E-11	0.38	0.41
ALDH18A1	rs11188403	0.29	0.42	3.5E-09	0.32	0.64
ALDH18A1	rs11188411	0.35	0.09	3.0E-08	0.35	0.98
ALDH18A1	rs12773566	0.22	0.16	0.07	0.26	0.39
ALDH18A1	rs12778814	0.45	0.12	1.9E-18	0.48	0.63
ALDH18A1	rs1409709	0.48	0.16	2.2E-10	0.46	0.74
ALDH18A1	rs2275272	0.13	0.05	0.06	0.11	0.67
ALDH18A1	rs3750701	0.34	0.42	0.23	0.31	0.44
ALDH18A1	rs3793749	0.31	0.48	9.5E-04	0.31	0.91
ALDH18A1	rs4037	0.35	0.09	3.9E-08	0.35	1.00
ALDH18A1	rs749049	0.50	0.41	0.23	0.48	0.71
ALDH18A1	rs8758	0.35	0.09	3.0E-08	NA	
ALDH18A1	rs945958	0.15	0.41	1.9E-09	0.18	0.29
ALDH1A1	rs348483	0.16	0.07	0.02	0.14	0.66
ALDH1A1	rs348484	0.16	0.07	0.02	0.14	0.66
ALDH1A1	rs348485	0.22	0.22	1.00	0.22	0.94
ALDH1A2	rs10518961	0.41	0.40	0.05	0.43	0.11
ALDH1A2	rs1063666	0.48	0.39	4.2E-03	0.45	0.70
ALDH1A2	rs12595180	0.48	0.40	2.9E-03	0.46	0.15
ALDH1A2	rs12903202	0.10	0.00	1.2E-04	0.11	0.73
ALDH1A2	rs1874158	0.08	0.19	0.002	0.11	0.22
ALDH1A2	rs2899613	0.44	0.41	5.6E-04	0.43	0.79
ALDH1A2	rs3204689	0.44	0.20	5.7E-06	0.46	0.06
ALDH1A2	rs3742959	0.48	0.37	1.6E-03	0.45	0.63
ALDH1A2	rs3784259	0.49	0.38	0.01	0.45	0.36
ALDH1A2	rs4646576	0.34	0.16	2.4E-04	0.36	0.65
ALDH1A2	rs4646615	0.34	0.26	0.18	0.36	0.01
ALDH1A2	rs4646626	0.49	0.40	0.02	0.45	0.36
ALDH1A2	rs4646642	0.48	0.39	0.01	0.47	0.89
ALDH1A2	rs4646644	0.08	0.19	0.001	0.12	0.08
ALDH1A2	rs4646645	0.47	0.38	0.01	0.45	0.73
ALDH1A3	rs1130738	0.45	0.46	0.71	0.43	0.90
ALDH1A3	rs11854028	0.46	0.44	0.88	0.44	0.58
ALDH1A3	rs14226	0.20	0.16	0.36	0.12	0.002
ALDH1A3	rs4646678	0.19	0.14	0.24	0.11	0.003
ALDH1A3	rs7182884	0.38	0.12	3.8E-07	0.49	0.003
ALDH1B1	rs10738997	0.27	0.47	1.9E-04	0.23	0.36
ALDH1B1	rs2073478	0.40	0.34	7.6E-06	0.39	0.49
ALDH1B1	rs3043	0.23	0.34	0.04	0.23	0.88
ALDH1B1	rs4242647	0.17	0.28	0.002	0.16	0.82
ALDH1B1	rs4878802	0.25	0.46	6.7E-05	0.22	0.51
ALDH1B1	rs4878803	0.17	0.38	6.1E-06	0.13	0.52
ALDH1B1	rs4878804	0.17	0.23	0.23	NA	
ALDH1B1	rs7040397	0.18	0.40	2.0E-06	0.17	0.94

ALDH1L1	rs11715574	0.17	0.36	1.6E-05	0.13	0.04
ALDH1L1	rs12636371	0.40	0.38	0.12	0.36	0.18
ALDH1L1	rs12638724	0.46	0.39	0.31	0.44	0.004
ALDH1L1	rs13060596	0.35	0.28	0.21	0.31	0.09
ALDH1L1	rs1868138	0.22	0.39	6.0E-04	0.20	0.40
ALDH1L1	rs2003334	0.35	0.36	0.96	0.33	0.82
ALDH1L1	rs2044576	0.05	0.15	0.001	NA	
ALDH1L1	rs2290053	0.46	0.45	0.95	0.50	0.07
ALDH1L1	rs3772411	0.30	0.16	0.002	0.29	0.94
ALDH1L1	rs3772426	0.31	0.22	0.13	0.30	0.84
ALDH1L1	rs3816366	0.37	0.34	0.88	0.35	0.11
ALDH1L1	rs3843355	0.48	0.42	0.09	0.43	0.32
ALDH1L1	rs4646715	0.35	0.48	0.01	0.39	0.59
ALDH1L1	rs4646717	0.35	0.48	0.01	0.36	0.57
ALDH1L1	rs4646739	0.44	0.37	0.25	0.42	0.33
ALDH1L1	rs4646745	0.24	0.14	0.03	0.16	0.001
ALDH1L1	rs4646755	0.26	0.10	4.8E-04	0.21	0.17
ALDH1L1	rs4646761	0.38	0.40	7.0E-05	0.35	0.75
ALDH1L1	rs6774437	0.49	0.45	0.39	0.46	0.10
ALDH1L1	rs7639966	0.42	0.28	0.01	0.43	0.41
ALDH1L1	rs9847790	0.18	0.32	0.001	0.12	0.13
ALDH1L2	rs10459204	0.16	0.08	0.05	0.17	0.81
ALDH1L2	rs10746009	0.37	0.45	0.29	0.37	0.28
ALDH1L2	rs10778364	0.47	0.28	3.2E-04	0.45	0.41
ALDH1L2	rs10861327	0.48	0.23	7.7E-06	0.50	0.75
ALDH1L2	rs12372293	0.12	0.01	9.1E-05	0.20	0.01
ALDH1L2	rs1345094	0.35	0.48	0.04	0.30	0.29
ALDH1L2	rs2374449	0.19	0.09	0.03	0.13	0.05
ALDH1L2	rs7299166	0.26	0.18	0.31	0.22	0.29
ALDH1L2	rs7954946	0.43	0.36	1.6E-04	0.39	0.38
ALDH2	rs10744777	0.35	0.11	4.4E-28	0.38	0.37
ALDH2	rs16941669	0.10	0.06	0.34	0.14	0.26
CYP1A2	rs2470890	0.38	0.13	7.0E-20	0.34	0.59
CYP1A2	rs2472304	0.38	0.13	4.8E-20	0.34	0.57
CYP1A2	rs762551	0.30	0.44	0.01	0.28	0.81
CYP1B1	rs1056836	0.42	0.40	1.7E-06	0.47	0.31
CYP1B1	rs1800440	0.21	0.05	5.6E-05	0.18	0.47
CYP1B1	rs2855658	0.42	0.40	9.5E-06	0.47	0.31
CYP2B6	rs7250601	0.22	0.32	0.04	0.28	0.08
CYP2C8	rs10509681	0.10	0.03	0.08	0.13	0.19
CYP2C8	rs1058932	0.19	0.36	3.2E-04	0.19	0.23
CYP2C8	rs11188156	0.33	0.44	0.03	0.32	0.70
CYP2C8	rs11572126	0.13	0.13	1.00	0.10	0.44
CYP2C8	rs11572174	0.10	0.03	0.08	0.13	0.19
CYP2C8	rs1891071	0.35	0.33	0.91	0.38	0.52
CYP2C8	rs1934952	0.35	0.28	0.20	0.35	0.99
CYP2C8	rs1934956	0.14	0.26	0.002	0.12	0.79
CYP2C8	rs2185571	0.32	0.13	9.8E-05	0.30	0.73
CYP2C8	rs2275622	0.32	0.26	0.46	0.33	0.82
CYP2C9	rs1057910	0.08	0.01	0.01	0.05	0.32
CYP2C9	rs1934963	0.18	0.15	0.32	0.20	0.87
CYP2C9	rs1934967	0.22	0.12	0.05	0.23	0.79
CYP2C9	rs1934968	0.09	0.13	0.18	0.10	0.60
CYP2C9	rs2298037	0.17	0.09	0.12	0.15	0.06
CYP2C9	rs4917636	0.11	0.03	0.02	0.16	0.10
CYP2C9	rs4918758	0.36	0.31	0.50	0.37	0.45
CYP2C9	rs9332100	0.11	0.03	0.02	0.15	0.30
CYP2C9	rs9332174	0.23	0.17	0.38	0.22	0.53

CYP2C9	rs9332216	0.11	0.03	0.02	0.15	0.34
CYP2C9	rs9332242	0.11	0.03	0.02	0.16	0.14
CYP2D6	rs1135840	0.43	0.36	0.30	NA	
CYP2E1	rs2070673	0.16	0.39	6.3E-24	0.16	0.86
CYP2E1	rs2515641	0.10	0.44	1.0E-14	0.13	0.08
CYP3A4	rs12333983	0.11	0.41	3.6E-23	0.12	0.47
ERCC2	rs13181	0.34	0.20	0.01	0.31	0.10
ERCC2	rs171140	0.49	0.18	5.8E-09	0.48	0.42
ERCC2	rs1799787	0.26	0.10	8.2E-04	0.25	0.29
ERCC2	rs3916874	0.26	0.12	0.01	0.34	0.02
ERCC2	rs50871	0.49	0.16	1.7E-10	0.46	0.13
ERCC2	rs50872	0.22	0.19	0.77	0.30	0.02
ERCC5	rs1047768	0.40	0.39	0.91	0.42	0.65
ERCC5	rs17655	0.21	0.45	4.4E-07	0.27	0.19
ERCC5	rs2016073	0.21	0.29	0.03	0.20	0.52
ERCC5	rs2094258	0.16	0.22	0.18	0.18	0.22
ERCC5	rs3759500	0.24	0.18	0.14	0.22	0.70
ERCC5	rs4150351	0.19	0.03	3.7E-05	0.18	0.81
ERCC5	rs4150383	0.18	0.06	0.002	0.18	0.79
ERCC5	rs4150386	0.12	0.03	0.02	0.13	0.81
ERCC5	rs4743	0.45	0.23	1.5E-05	0.36	0.07
G6PC	rs161630	0.09	0.17	0.01	0.11	0.47
G6PC	rs2229611	0.23	0.38	2.0E-03	0.24	0.87
G6PC	rs324079	0.00	0.19	0.13	0.13	0.53
GSTM1	GSTM1	0.46	0.36	0.01		
GSTM1	rs3815029	0.40	0.34	0.07	NA	
GSTP1	rs1138272	0.09	0.02	0.01	0.09	0.58
GSTP1	rs1695	0.35	0.30	0.63	0.40	0.15
GSTP1	rs6591256	0.42	0.29	0.02	0.46	0.56
GSTT1	GSTT1	0.20	0.27	0.25		
HIF1A	rs10873142	0.16	0.43	1.5E-18	0.14	0.57
HIF1A	rs11549465	0.09	0.04	0.39	0.07	0.76
HIF1A	rs12434438	0.19	0.32	1.5E-23	0.17	0.46
HIF1A	rs2057482	0.13	0.33	6.0E-06	0.10	0.38
HIF1A	rs2301113	0.21	0.27	1.4E-25	0.18	0.48
HNF4A	rs11086926	0.08	0.21	2.9E-04	0.05	0.17
HNF4A	rs11574736	0.18	0.08	0.02	0.17	0.90
HNF4A	rs2071200	0.26	0.49	5.4E-09	0.22	0.39
HNF4A	rs2425640	0.37	0.38	0.81	0.30	0.11
HNF4A	rs3212199	0.40	0.41	4.8E-04	0.43	0.64
HNF4A	rs3818247	0.37	0.47	1.6E-03	0.34	0.30
HNF4A	rs6031595	0.48	0.20	9.0E-10	0.44	0.52
HNF4A	rs6073432	0.31	0.32	0.83	0.30	0.77
HNF4A	rs6093978	0.35	0.29	0.47	0.35	0.93
HNF4A	rs6130615	0.12	0.49	3.2E-19	0.13	0.69
HNF4A	rs8114057	0.48	0.50	0.16	0.47	0.78
MDM2	rs1625525	0.41	0.20	4.6E-05	0.49	0.05
MDM2	rs1846402	0.19	0.09	0.01	0.18	0.87
MDM2	rs3730581	0.37	0.25	0.06	0.28	0.02
NAT2	rs11780272	0.43	0.28	0.01	0.41	0.88
NAT2	rs1208	0.42	0.39	0.63	0.41	0.93
NAT2	rs17516895	0.34	0.21	0.02	NA	
NAT2	rs1801280	0.45	0.33	0.07	0.47	0.73
NAT2	rs1961456	0.30	0.37	0.08	0.31	0.39
NAT2	rs4646242	0.29	0.22	0.43	0.31	0.43
NAT2	rs4646243	0.16	0.18	0.30	0.19	0.04
NAT2	rs4646246	0.19	0.28	0.03	0.22	0.09
NAT2	rs6984200	0.29	0.27	0.77	0.31	0.67

NAT2	rs7832071	0.42	0.40	0.36	0.40	0.88
NFE2L2	rs13401026	0.10	0.22	2.8E-04	0.11	0.95
NFE2L2	rs2588882	0.08	0.10	0.23	0.08	0.68
NFE2L2	rs8470	0.19	0.46	1.2E-12	0.21	0.61
NFE2L2	rs9068	0.19	0.50	1.3E-14	0.21	0.61
NOS2A	rs10459953	0.36	0.33	0.15	0.44	0.16
NOS2A	rs1113283	0.22	0.22	0.94	0.22	0.19
NOS2A	rs2297516	0.40	0.41	0.89	0.39	0.62
NOS2A	rs2297518	0.18	0.13	0.35	0.17	0.73
NOS2A	rs2779252	0.05	0.10	0.05	0.05	0.82
NOS2A	rs3794763	0.22	0.29	0.01	0.19	0.50
NOS2A	rs4795067	0.33	0.29	0.73	0.33	0.42
NOS2A	rs4796052	0.19	0.16	0.80	0.16	0.33
NOS2A	rs8072199	0.42	0.19	1.0E-05	0.48	0.03
NOS2A	rs944725	0.38	0.47	0.21	0.38	0.55
NQO1	rs10517	0.16	0.21	0.21	0.10	0.04
NQO1	rs17298718	0.08	0.04	0.58	0.07	1.00
NQO1	rs1800566	0.19	0.32	3.2E-04	0.18	0.79
NQO1	rs34763938	0.06	0.07	0.24	NA	
PTGS2	rs10911902	0.17	0.07	0.03	0.22	0.06
PTGS2	rs5277	0.13	0.06	0.03	0.19	0.12
RAD23B	rs10868	0.12	0.02	2.4E-04	0.11	0.61
RAD23B	rs1805329	0.21	0.05	7.6E-05	0.14	0.16
RAD23B	rs1805330	0.07	0.16	0.01	0.09	0.27
RAD23B	rs1805334	0.26	0.09	4.6E-04	0.23	0.57
RAD23B	rs7041137	0.29	0.32	0.36	0.26	0.58
SULT1A1	rs1968752	0.40	0.34	0.58	0.36	0.56
TP53	rs1614984	0.43	0.34	0.02	NA	
XPA	rs1800975	0.34	0.32	0.34	0.37	0.45
XPA	rs3176757	0.21	0.23	0.30	0.25	0.42
XPC	rs1043943	0.41	0.34	0.38	0.39	0.85
XPC	rs10468	0.21	0.05	5.6E-05	0.29	0.04
XPC	rs1106087	0.22	0.10	0.02	0.28	0.06
XPC	rs2228000	0.22	0.10	0.02	0.28	0.07
XPC	rs2228001	0.40	0.34	0.39	0.40	0.99
XPC	rs2470458	0.19	0.10	0.002	0.25	0.09
XPC	rs2607737	0.49	0.46	0.78	0.48	0.95
XPC	rs2607772	0.40	0.32	0.20	0.40	0.96
XPC	rs2733534	0.47	0.24	1.5E-05	0.48	0.98
XPC	rs2733537	0.29	0.19	0.07	0.37	0.04
XPC	rs2733587	0.10	0.09	0.88	0.16	0.04
XPC	rs3731149	0.26	0.19	0.26	0.20	0.06
