# Supplementary material for: Gastrointestinal Stromal Tumors, Somatic Mutations and Candidate Genetic Risk Variants
Source: PLoS One. 2013 Apr 18;8(4):e62119. doi: 10.1371/journal.pone.0062119 (PMC3630216; doi:10.1371/journal.pone.0062119)
Supplement: Table S2 — Minor allele frequencies (MAF), Odds Ratios (ORs) and association p-values by mutation type. (PDF) [file pone.0062119.s002.pdf]

Supplementary Table S2: Minor allele frequencies (MAF), Odds Ratios (ORs) and association p-values by mutation type									
**Gene**	**SNP**		**KIT exon 11 codon 557-8 deletion**	**KIT exon 11 insertion**	**KIT exon 11 other deletion**	**KIT exon 11 point mutation**	**Other KIT mutation**	**PDGFRA mutation**	**Wild type**
ADH1A	rs12508502	**MAF[a]**	0.09/0.09	0.09/0.09	0.04/0.10	0.10/0.09	0.17/0.08	0.03/0.10	0.14/0.08
		**OR (95% CI)**	1.05 (0.52, 2.13)	1.00 (0.37, 2.71)	0.43 (0.15, 1.25)	1.19 (0.57, 2.48)	2.12 (0.78, 5.76)	3.22 (0.74, 13.97)	1.85 (0.81, 4.23)
		**p-value**	0.9	1.0	0.1	0.6	0.1	0.1	0.1
ADH1B	rs1042026	**MAF[a]**	0.34/0.25	0.34/0.26	0.26/0.28	0.28/0.27	0.25/0.27	0.17/0.28	0.19/0.28
		**OR (95% CI)**	1.48 (0.99, 2.22)	1.33 (0.76, 2.34)	0.90 (0.55, 1.48)	1.00 (0.64, 1.56)	0.90 (0.42, 1.90)	1.87 (0.92, 3.77)	0.63 (0.34, 1.17)
		**p-value**	0.1	0.3	0.7	1.0	0.8	0.1	0.1
ADH1B	rs17033	**MAF[a]**	0.08/0.09	0.09/0.09	0.04/0.10	0.11/0.09	0.17/0.09	0.07/0.09	0.13/0.09
		**OR (95% CI)**	0.90 (0.44, 1.86)	1.00 (0.37, 2.70)	0.42 (0.14, 1.22)	1.31 (0.64, 2.69)	2.08 (0.77, 5.64)	1.46 (0.49, 4.34)	1.41 (0.61, 3.26)
		**p-value**	0.8	1.0	0.1	0.5	0.1	0.5	0.4
ADH1C	rs1693482	**MAF[a]**	0.27/0.38	0.27/0.36	0.39/0.34	0.38/0.34	0.28/0.36	0.41/0.34	0.46/0.34
		**OR (95% CI)**	0.63 (0.40, 0.98)	0.65 (0.35, 1.21)	1.35 (0.83, 2.19)	1.24 (0.80, 1.91)	0.60 (0.29, 1.27)	0.77 (0.43, 1.37)	1.47 (0.88, 2.46)
		**p-value**	0.0	0.2	0.2	0.3	0.2	0.4	0.1
AHR	rs2066853	**MAF[a]**	0.19/0.18	0.18/0.19	0.17/0.19	0.20/0.18	0.08/0.19	0.17/0.19	0.25/0.17
		**OR (95% CI)**	0.91 (0.53, 1.54)	1.02 (0.48, 2.15)	0.74 (0.39, 1.39)	1.10 (0.63, 1.92)	0.48 (0.14, 1.65)	0.97 (0.46, 2.01)	1.94 (1.03, 3.69)
		**p-value**	0.7	1.0	0.3	0.7	0.2	0.9	0.0
AHR	rs713150	**MAF[a]**	0.21/0.25	0.29/0.24	0.24/0.24	0.21/0.25	0.19/0.24	0.28/0.24	0.26/0.24
		**OR (95% CI)**	0.82 (0.51, 1.32)	1.34 (0.72, 2.50)	1.06 (0.62, 1.81)	0.82 (0.49, 1.36)	0.71 (0.30, 1.69)	0.77 (0.41, 1.44)	1.11 (0.61, 2.03)
		**p-value**	0.4	0.4	0.8	0.4	0.4	0.4	0.7
ALDH18A1	rs10509688	**MAF[a]**	0.14/0.16	0.16/0.16	0.17/0.15	0.19/0.15	0.11/0.16	0.10/0.16	0.18/0.15
		**OR (95% CI)**	0.82 (0.47, 1.43)	1.07 (0.50, 2.28)	1.12 (0.61, 2.05)	1.33 (0.77, 2.30)	0.63 (0.21, 1.86)	1.65 (0.67, 4.05)	1.12 (0.57, 2.22)
		**p-value**	0.5	0.9	0.7	0.3	0.4	0.3	0.7
ALDH18A1	rs1053905	**MAF[a]**	0.48/0.48	0.54/0.48	0.38/0.50	0.46/0.49	0.58/0.48	0.48/0.48	0.56/0.47
		**OR (95% CI)**	1.09 (0.71, 1.68)	1.35 (0.72, 2.51)	0.62 (0.37, 1.04)	0.92 (0.58, 1.47)	1.30 (0.63, 2.71)	1.07 (0.58, 1.97)	1.22 (0.71, 2.11)
		**p-value**	0.7	0.3	0.1	0.7	0.5	0.8	0.5
ALDH18A1	rs11188388	**MAF[a]**	0.07/0.08	0.05/0.08	0.09/0.07	0.08/0.08	0.00/0.08	0.14/0.07	0.08/0.08
		**OR (95% CI)**	0.84 (0.39, 1.80)	0.66 (0.20, 2.23)	1.20 (0.54, 2.69)	1.07 (0.50, 2.31)	0.00 (0.00, 0.00)	0.46 (0.20, 1.07)	1.12 (0.46, 2.74)
		**p-value**	0.7	0.5	0.7	0.9	1.0	0.1	0.8
ALDH18A1	rs11188394	**MAF[a]**	0.32/0.28	0.30/0.29	0.21/0.31	0.28/0.30	0.33/0.29	0.33/0.29	0.32/0.29
		**OR (95% CI)**	1.33 (0.85, 2.09)	1.05 (0.55, 2.00)	0.65 (0.37, 1.16)	0.88 (0.53, 1.44)	1.03 (0.49, 2.16)	0.81 (0.44, 1.49)	1.05 (0.60, 1.84)
		**p-value**	0.2	0.9	0.1	0.6	0.9	0.5	0.9
ALDH18A1	rs11188403	**MAF[a]**	0.31/0.35	0.29/0.35	0.42/0.32	0.36/0.34	0.28/0.34	0.38/0.34	0.31/0.35
		**OR (95% CI)**	0.76 (0.49, 1.18)	0.73 (0.39, 1.40)	1.42 (0.87, 2.33)	1.08 (0.68, 1.69)	0.86 (0.40, 1.85)	0.73 (0.39, 1.35)	0.93 (0.53, 1.62)
		**p-value**	0.2	0.3	0.2	0.8	0.7	0.3	0.8
ALDH18A1	rs11188411	**MAF[a]**	0.33/0.30	0.36/0.30	0.21/0.32	0.28/0.31	0.33/0.30	0.33/0.30	0.36/0.30
		**OR (95% CI)**	1.29 (0.84, 2.00)	1.32 (0.72, 2.42)	0.60 (0.34, 1.05)	0.81 (0.50, 1.31)	0.98 (0.47, 2.04)	0.87 (0.48, 1.59)	1.20 (0.69, 2.07)
		**p-value**	0.2	0.4	0.1	0.4	0.9	0.7	0.5
ALDH18A1	rs12773566	**MAF[a]**	0.16/0.22	0.23/0.21	0.20/0.21	0.21/0.21	0.19/0.21	0.22/0.21	0.25/0.20
		**OR (95% CI)**	0.69 (0.42, 1.15)	1.19 (0.62, 2.28)	0.99 (0.57, 1.71)	1.07 (0.65, 1.76)	0.85 (0.36, 1.97)	0.91 (0.48, 1.75)	1.19 (0.67, 2.13)
		**p-value**	0.2	0.6	1.0	0.8	0.7	0.8	0.6
ALDH18A1	rs12778814	**MAF[a]**	0.46/0.47	0.48/0.47	0.38/0.49	0.46/0.47	0.58/0.46	0.48/0.47	0.51/0.46
		**OR (95% CI)**	1.11 (0.71, 1.72)	1.07 (0.57, 2.01)	0.67 (0.40, 1.14)	1.01 (0.63, 1.61)	1.38 (0.66, 2.89)	1.01 (0.54, 1.87)	1.08 (0.63, 1.86)

		**p-value**	0.7	0.8	0.1	1.0	0.4	1.0	0.8
ALDH18A1	rs1409709	**MAF[a]**	0.45/0.42	0.44/0.42	0.33/0.44	0.43/0.42	0.50/0.42	0.36/0.43	0.49/0.41
		**OR (95% CI)**	1.27 (0.83, 1.94)	1.13 (0.61, 2.09)	0.67 (0.40, 1.12)	1.05 (0.67, 1.65)	1.19 (0.59, 2.40)	1.43 (0.78, 2.61)	1.16 (0.69, 1.96)
		**p-value**	0.3	0.7	0.1	0.8	0.6	0.3	0.6
ALDH18A1	rs2275272	**MAF[a]**	0.11/0.11	0.09/0.11	0.11/0.11	0.14/0.10	0.17/0.11	0.03/0.12	0.11/0.11
		**OR (95% CI)**	1.00 (0.53, 1.91)	0.77 (0.29, 2.04)	1.07 (0.51, 2.24)	1.60 (0.85, 3.03)	1.57 (0.61, 4.02)	4.39 (1.02, 18.90)	0.90 (0.40, 2.05)
		**p-value**	1.0	0.6	0.9	0.1	0.4	0.0	0.8
ALDH18A1	rs3750701	**MAF[a]**	0.36/0.35	0.29/0.36	0.34/0.35	0.35/0.35	0.39/0.35	0.45/0.34	0.31/0.36
		**OR (95% CI)**	1.04 (0.70, 1.54)	0.73 (0.40, 1.33)	0.92 (0.58, 1.46)	1.01 (0.66, 1.56)	1.24 (0.62, 2.46)	0.69 (0.41, 1.18)	0.82 (0.48, 1.41)
		**p-value**	0.9	0.3	0.7	1.0	0.5	0.2	0.5
ALDH18A1	rs3793749	**MAF[a]**	0.30/0.35	0.36/0.34	0.44/0.32	0.38/0.33	0.28/0.34	0.22/0.35	0.33/0.34
		**OR (95% CI)**	0.76 (0.50, 1.16)	1.07 (0.59, 1.94)	1.61 (1.01, 2.57)	1.19 (0.76, 1.85)	0.80 (0.37, 1.71)	1.83 (0.96, 3.50)	1.03 (0.60, 1.77)
		**p-value**	0.2	0.8	0.0	0.4	0.6	0.1	0.9
ALDH18A1	rs4037	**MAF[a]**	0.33/0.30	0.36/0.30	0.21/0.32	0.28/0.31	0.33/0.30	0.33/0.30	0.36/0.30
		**OR (95% CI)**	1.29 (0.84, 2.00)	1.32 (0.72, 2.42)	0.60 (0.34, 1.05)	0.81 (0.50, 1.31)	0.98 (0.47, 2.04)	0.87 (0.48, 1.59)	1.20 (0.69, 2.07)
		**p-value**	0.2	0.4	0.1	0.4	0.9	0.7	0.5
ALDH18A1	rs749049	**MAF[a]**	0.50/0.49	0.55/0.48	0.49/0.49	0.46/0.50	0.50/0.49	0.45/0.50	0.51/0.49
		**OR (95% CI)**	1.07 (0.72, 1.58)	1.29 (0.74, 2.25)	1.02 (0.65, 1.60)	0.84 (0.55, 1.28)	1.01 (0.51, 1.98)	1.17 (0.68, 2.01)	1.12 (0.68, 1.87)
		**p-value**	0.7	0.4	0.9	0.4	1.0	0.6	0.7
ALDH18A1	rs8758	**MAF[a]**	0.33/0.30	0.36/0.30	0.21/0.32	0.28/0.31	0.33/0.30	0.33/0.30	0.36/0.30
		**OR (95% CI)**	1.29 (0.84, 2.00)	1.32 (0.72, 2.42)	0.60 (0.34, 1.05)	0.81 (0.50, 1.31)	0.98 (0.47, 2.04)	0.87 (0.48, 1.59)	1.20 (0.69, 2.07)
		**p-value**	0.2	0.4	0.1	0.4	0.9	0.7	0.5
ALDH18A1	rs945958	**MAF[a]**	0.24/0.19	0.14/0.21	0.20/0.20	0.18/0.20	0.17/0.20	0.29/0.19	0.14/0.21
		**OR (95% CI)**	1.26 (0.79, 2.00)	0.65 (0.30, 1.43)	0.88 (0.50, 1.55)	0.84 (0.49, 1.44)	1.01 (0.42, 2.46)	0.53 (0.28, 0.99)	0.80 (0.40, 1.60)
		**p-value**	0.3	0.3	0.7	0.5	1.0	0.0	0.5
ALDH1A1	rs348483	**MAF[a]**	0.13/0.15	0.13/0.15	0.14/0.14	0.15/0.14	0.22/0.14	0.14/0.14	0.14/0.14
		**OR (95% CI)**	0.89 (0.51, 1.57)	0.79 (0.35, 1.81)	1.06 (0.56, 1.98)	1.05 (0.59, 1.87)	1.63 (0.73, 3.65)	1.07 (0.49, 2.34)	0.92 (0.45, 1.89)
		**p-value**	0.7	0.6	0.9	0.9	0.2	0.9	0.8
ALDH1A1	rs348484	**MAF[a]**	0.13/0.15	0.13/0.15	0.14/0.14	0.15/0.14	0.22/0.14	0.14/0.14	0.14/0.14
		**OR (95% CI)**	0.89 (0.51, 1.57)	0.79 (0.35, 1.81)	1.06 (0.56, 1.98)	1.05 (0.59, 1.87)	1.63 (0.73, 3.65)	1.07 (0.49, 2.34)	0.92 (0.45, 1.89)
		**p-value**	0.7	0.6	0.9	0.9	0.2	0.9	0.8
ALDH1A1	rs348485	**MAF[a]**	0.25/0.21	0.20/0.22	0.23/0.22	0.22/0.22	0.22/0.22	0.21/0.22	0.19/0.23
		**OR (95% CI)**	1.21 (0.77, 1.92)	0.82 (0.41, 1.64)	1.06 (0.62, 1.82)	0.97 (0.59, 1.61)	1.03 (0.46, 2.34)	1.07 (0.54, 2.10)	0.84 (0.44, 1.60)
		**p-value**	0.4	0.6	0.8	0.9	0.9	0.9	0.6
ALDH1A2	rs10518961	**MAF[a]**	0.45/0.40	0.43/0.41	0.33/0.43	0.39/0.41	0.44/0.41	0.34/0.42	0.49/0.40
		**OR (95% CI)**	1.20 (0.82, 1.75)	1.08 (0.63, 1.85)	0.70 (0.44, 1.11)	0.90 (0.60, 1.35)	1.16 (0.59, 2.28)	1.29 (0.73, 2.30)	1.41 (0.85, 2.36)
		**p-value**	0.4	0.8	0.1	0.6	0.7	0.4	0.2
ALDH1A2	rs1063666	**MAF[a]**	0.46/0.48	0.54/0.49	0.47/0.50	0.48/0.50	0.47/0.50	0.41/0.49	0.53/0.49
		**OR (95% CI)**	1.27 (0.85, 1.88)	1.19 (0.69, 2.08)	0.92 (0.59, 1.45)	0.90 (0.60, 1.37)	0.83 (0.41, 1.67)	1.39 (0.78, 2.46)	1.06 (0.63, 1.79)
		**p-value**	0.2	0.5	0.7	0.6	0.6	0.3	0.8
ALDH1A2	rs12595180	**MAF[a]**	0.47/0.49	0.57/0.49	0.47/0.50	0.45/0.51	0.50/0.50	0.43/0.50	0.54/0.49
		**OR (95% CI)**	1.21 (0.83, 1.78)	1.37 (0.79, 2.38)	0.92 (0.59, 1.43)	0.75 (0.50, 1.14)	0.96 (0.48, 1.90)	1.26 (0.72, 2.20)	1.15 (0.69, 1.93)
		**p-value**	0.3	0.3	0.7	0.2	0.9	0.4	0.6
ALDH1A2	rs12903202	**MAF[a]**	0.08/0.08	0.09/0.07	0.12/0.07	0.05/0.08	0.03/0.08	0.07/0.08	0.07/0.08
		**OR (95% CI)**	1.10 (0.51, 2.38)	1.27 (0.45, 3.56)	2.44 (1.11, 5.37)	0.64 (0.25, 1.59)	0.27 (0.03, 2.04)	1.13 (0.37, 3.47)	0.70 (0.25, 1.96)

		**p-value**	0.8	0.6	0.0	0.3	0.2	0.8	0.5
ALDH1A2	rs1874158	**MAF[a]**	0.11/0.11	0.16/0.10	0.12/0.10	0.11/0.11	0.08/0.11	0.05/0.11	0.10/0.11
		**OR (95% CI)**	0.93 (0.48, 1.80)	1.80 (0.78, 4.14)	1.13 (0.54, 2.35)	0.95 (0.47, 1.94)	0.90 (0.25, 3.24)	2.26 (0.66, 7.71)	1.11 (0.45, 2.74)
		**p-value**	0.8	0.2	0.7	0.9	0.9	0.2	0.8
ALDH1A2	rs2899613	**MAF[a]**	0.43/0.48	0.43/0.47	0.49/0.46	0.53/0.45	0.47/0.47	0.48/0.46	0.42/0.47
		**OR (95% CI)**	0.81 (0.55, 1.20)	0.86 (0.50, 1.49)	1.05 (0.68, 1.64)	1.39 (0.92, 2.09)	1.11 (0.56, 2.20)	0.97 (0.56, 1.68)	0.85 (0.50, 1.42)
		**p-value**	0.3	0.6	0.8	0.1	0.8	0.9	0.5
ALDH1A2	rs3204689	**MAF[a]**	0.42/0.38	0.39/0.39	0.37/0.40	0.35/0.40	0.42/0.39	0.38/0.39	0.44/0.38
		**OR (95% CI)**	1.22 (0.81, 1.83)	1.01 (0.57, 1.78)	0.97 (0.60, 1.55)	0.78 (0.50, 1.20)	0.96 (0.48, 1.93)	1.03 (0.58, 1.84)	1.10 (0.65, 1.85)
		**p-value**	0.3	1.0	0.9	0.3	0.9	0.9	0.7
ALDH1A2	rs3742959	**MAF[a]**	0.45/0.48	0.54/0.49	0.44/0.50	0.46/0.50	0.47/0.49	0.43/0.50	0.53/0.49
		**OR (95% CI)**	1.35 (0.91, 1.99)	1.20 (0.70, 2.07)	0.85 (0.54, 1.33)	0.84 (0.56, 1.27)	0.84 (0.42, 1.67)	1.25 (0.72, 2.19)	1.06 (0.64, 1.78)
		**p-value**	0.1	0.5	0.5	0.4	0.6	0.4	0.8
ALDH1A2	rs3784259	**MAF[a]**	0.48/0.47	0.54/0.48	0.44/0.49	0.45/0.49	0.50/0.48	0.40/0.49	0.53/0.48
		**OR (95% CI)**	1.25 (0.85, 1.84)	1.26 (0.73, 2.16)	0.89 (0.57, 1.38)	0.82 (0.55, 1.24)	1.01 (0.51, 1.98)	1.40 (0.80, 2.46)	1.12 (0.68, 1.86)
		**p-value**	0.2	0.4	0.6	0.3	1.0	0.2	0.7
ALDH1A2	rs4646576	**MAF[a]**	0.31/0.30	0.29/0.30	0.22/0.32	0.28/0.31	0.39/0.30	0.31/0.30	0.39/0.29
		**OR (95% CI)**	1.11 (0.72, 1.71)	0.91 (0.49, 1.69)	0.65 (0.37, 1.12)	0.84 (0.53, 1.35)	1.36 (0.66, 2.80)	0.94 (0.51, 1.74)	1.43 (0.83, 2.47)
		**p-value**	0.6	0.8	0.1	0.5	0.4	0.8	0.2
ALDH1A2	rs4646615	**MAF[a]**	0.34/0.31	0.32/0.32	0.34/0.31	0.30/0.32	0.28/0.32	0.26/0.32	0.33/0.31
		**OR (95% CI)**	1.17 (0.77, 1.78)	1.04 (0.57, 1.88)	1.20 (0.74, 1.94)	0.93 (0.59, 1.46)	0.77 (0.36, 1.66)	1.38 (0.73, 2.60)	1.00 (0.57, 1.73)
		**p-value**	0.5	0.9	0.5	0.7	0.5	0.3	1.0
ALDH1A2	rs4646626	**MAF[a]**	0.48/0.47	0.54/0.48	0.46/0.49	0.45/0.49	0.50/0.48	0.40/0.49	0.53/0.48
		**OR (95% CI)**	1.23 (0.84, 1.81)	1.25 (0.73, 2.15)	0.92 (0.59, 1.43)	0.82 (0.55, 1.23)	1.01 (0.51, 1.97)	1.41 (0.80, 2.47)	1.11 (0.67, 1.84)
		**p-value**	0.3	0.4	0.7	0.3	1.0	0.2	0.7
ALDH1A2	rs4646642	**MAF[a]**	0.46/0.49	0.54/0.49	0.47/0.50	0.49/0.50	0.47/0.50	0.41/0.49	0.53/0.49
		**OR (95% CI)**	0.79 (0.53, 1.17)	0.84 (0.48, 1.47)	1.10 (0.70, 1.73)	1.07 (0.71, 1.62)	1.22 (0.61, 2.44)	0.72 (0.40, 1.27)	0.96 (0.57, 1.63)
		**p-value**	0.2	0.5	0.7	0.8	0.6	0.3	0.9
ALDH1A2	rs4646644	**MAF[a]**	0.12/0.10	0.14/0.10	0.10/0.10	0.13/0.10	0.06/0.11	0.03/0.11	0.08/0.11
		**OR (95% CI)**	1.18 (0.62, 2.25)	1.58 (0.66, 3.79)	0.85 (0.39, 1.86)	1.44 (0.73, 2.84)	0.57 (0.13, 2.56)	3.37 (0.79, 14.46)	0.88 (0.34, 2.29)
		**p-value**	0.6	0.3	0.7	0.3	0.5	0.1	0.8
ALDH1A2	rs4646645	**MAF[a]**	0.45/0.48	0.54/0.49	0.47/0.50	0.47/0.50	0.47/0.50	0.43/0.49	0.53/0.49
		**OR (95% CI)**	0.76 (0.51, 1.12)	0.84 (0.48, 1.46)	1.10 (0.70, 1.72)	1.16 (0.77, 1.76)	1.22 (0.61, 2.45)	0.77 (0.44, 1.37)	0.97 (0.58, 1.63)
		**p-value**	0.2	0.5	0.7	0.5	0.6	0.4	0.9
ALDH1A3	rs1130738	**MAF[a]**	0.45/0.46	0.36/0.46	0.52/0.44	0.46/0.45	0.58/0.44	0.43/0.46	0.40/0.46
		**OR (95% CI)**	0.97 (0.66, 1.42)	0.64 (0.36, 1.14)	1.38 (0.88, 2.16)	1.05 (0.69, 1.60)	1.77 (0.88, 3.54)	1.11 (0.64, 1.91)	0.79 (0.47, 1.32)
		**p-value**	0.9	0.1	0.2	0.8	0.1	0.7	0.4
ALDH1A3	rs11854028	**MAF[a]**	0.45/0.46	0.34/0.47	0.54/0.44	0.46/0.46	0.56/0.45	0.47/0.46	0.40/0.47
		**OR (95% CI)**	0.99 (0.68, 1.46)	0.58 (0.32, 1.04)	1.51 (0.96, 2.37)	1.00 (0.66, 1.51)	1.50 (0.76, 2.93)	0.98 (0.57, 1.67)	0.78 (0.47, 1.30)
		**p-value**	1.0	0.1	0.1	1.0	0.2	0.9	0.3
ALDH1A3	rs14226	**MAF[a]**	0.21/0.19	0.21/0.19	0.19/0.19	0.19/0.19	0.17/0.19	0.19/0.19	0.17/0.20
		**OR (95% CI)**	1.22 (0.75, 2.00)	1.17 (0.60, 2.30)	1.02 (0.57, 1.82)	0.97 (0.57, 1.66)	0.76 (0.30, 1.93)	1.02 (0.51, 2.06)	0.71 (0.35, 1.46)
		**p-value**	0.4	0.6	0.9	0.9	0.6	0.9	0.4
ALDH1A3	rs4646678	**MAF[a]**	0.20/0.18	0.20/0.18	0.18/0.19	0.19/0.18	0.17/0.19	0.17/0.19	0.18/0.19
		**OR (95% CI)**	1.15 (0.70, 1.87)	1.08 (0.54, 2.14)	0.99 (0.55, 1.77)	1.03 (0.60, 1.74)	0.81 (0.33, 2.01)	1.11 (0.54, 2.26)	0.87 (0.44, 1.70)
		**p-value**	0.6	0.8	1.0	0.9	0.6	0.8	0.7

ALDH1A3	rs7182884	**MAF**[a]	0.31/0.33	0.36/0.32	0.27/0.33	0.30/0.33	0.25/0.33	0.38/0.32	0.39/0.31
		**OR (95% CI)**	1.01 (0.66, 1.53)	1.19 (0.67, 2.12)	0.80 (0.49, 1.33)	0.92 (0.59, 1.45)	0.59 (0.27, 1.28)	0.81 (0.47, 1.40)	1.25 (0.74, 2.12)
		**p-value**	1.0	0.6	0.4	0.7	0.2	0.4	0.4
ALDH1B1	rs10738997	**MAF**[a]	0.33/0.31	0.36/0.31	0.27/0.32	0.33/0.31	0.22/0.32	0.33/0.31	0.33/0.31
		**OR (95% CI)**	1.05 (0.70, 1.58)	1.21 (0.69, 2.13)	0.71 (0.43, 1.18)	1.04 (0.68, 1.61)	0.70 (0.32, 1.54)	0.88 (0.49, 1.57)	1.27 (0.75, 2.14)
		**p-value**	0.8	0.5	0.2	0.8	0.4	0.7	0.4
ALDH1B1	rs2073478	**MAF**[a]	0.50/0.45	0.48/0.46	0.39/0.47	0.41/0.47	0.47/0.46	0.48/0.45	0.47/0.46
		**OR (95% CI)**	1.16 (0.79, 1.69)	1.13 (0.66, 1.93)	0.66 (0.42, 1.06)	0.77 (0.51, 1.18)	1.15 (0.60, 2.20)	0.74 (0.43, 1.27)	1.09 (0.67, 1.77)
		**p-value**	0.5	0.7	0.1	0.2	0.7	0.3	0.7
ALDH1B1	rs3043	**MAF**[a]	0.29/0.26	0.29/0.26	0.21/0.28	0.21/0.28	0.28/0.26	0.34/0.26	0.28/0.26
		**OR (95% CI)**	1.11 (0.72, 1.71)	1.17 (0.63, 2.16)	0.66 (0.38, 1.14)	0.68 (0.41, 1.13)	1.17 (0.55, 2.48)	0.64 (0.35, 1.15)	1.12 (0.63, 1.97)
		**p-value**	0.6	0.6	0.1	0.1	0.7	0.1	0.7
ALDH1B1	rs4242647	**MAF**[a]	0.20/0.19	0.21/0.19	0.20/0.19	0.22/0.18	0.14/0.20	0.17/0.19	0.15/0.20
		**OR (95% CI)**	1.00 (0.61, 1.65)	1.12 (0.57, 2.21)	1.01 (0.57, 1.80)	1.20 (0.72, 2.01)	0.75 (0.29, 1.96)	1.09 (0.53, 2.26)	0.86 (0.43, 1.72)
		**p-value**	1.0	0.7	1.0	0.5	0.6	0.8	0.7
ALDH1B1	rs4878802	**MAF**[a]	0.32/0.29	0.36/0.29	0.26/0.30	0.30/0.29	0.19/0.30	0.31/0.29	0.29/0.30
		**OR (95% CI)**	1.08 (0.71, 1.65)	1.36 (0.76, 2.44)	0.74 (0.44, 1.24)	1.00 (0.64, 1.56)	0.65 (0.28, 1.49)	0.83 (0.45, 1.52)	1.11 (0.64, 1.92)
		**p-value**	0.7	0.3	0.3	1.0	0.3	0.5	0.7
ALDH1B1	rs4878803	**MAF**[a]	0.23/0.20	0.23/0.21	0.22/0.21	0.20/0.22	0.14/0.22	0.22/0.21	0.19/0.21
		**OR (95% CI)**	1.11 (0.69, 1.77)	1.12 (0.58, 2.18)	0.99 (0.57, 1.71)	0.85 (0.50, 1.43)	0.71 (0.27, 1.86)	0.85 (0.44, 1.66)	1.10 (0.58, 2.07)
		**p-value**	0.7	0.7	1.0	0.5	0.5	0.6	0.8
ALDH1B1	rs4878804	**MAF**[a]	0.19/0.18	0.23/0.18	0.17/0.18	0.19/0.18	0.14/0.18	0.17/0.18	0.17/0.18
		**OR (95% CI)**	1.05 (0.64, 1.73)	1.35 (0.70, 2.61)	0.87 (0.48, 1.58)	0.99 (0.58, 1.69)	0.78 (0.30, 2.03)	1.01 (0.49, 2.08)	1.02 (0.53, 1.97)
		**p-value**	0.8	0.4	0.6	1.0	0.6	1.0	1.0
ALDH1B1	rs7040397	**MAF**[a]	0.26/0.21	0.23/0.22	0.21/0.22	0.21/0.22	0.14/0.22	0.22/0.22	0.21/0.22
		**OR (95% CI)**	1.24 (0.79, 1.96)	1.08 (0.56, 2.09)	0.87 (0.50, 1.52)	0.87 (0.52, 1.45)	0.67 (0.26, 1.75)	0.90 (0.46, 1.75)	1.13 (0.62, 2.08)
		**p-value**	0.4	0.8	0.6	0.6	0.4	0.8	0.7
ALDH1L1	rs11715574	**MAF**[a]	0.20/0.21	0.29/0.20	0.22/0.21	0.24/0.20	0.11/0.22	0.22/0.21	0.15/0.22
		**OR (95% CI)**	0.84 (0.51, 1.38)	1.53 (0.80, 2.91)	0.99 (0.57, 1.73)	1.17 (0.70, 1.96)	0.54 (0.18, 1.58)	0.85 (0.44, 1.65)	0.84 (0.41, 1.70)
		**p-value**	0.5	0.2	1.0	0.5	0.3	0.6	0.6
ALDH1L1	rs12636371	**MAF**[a]	0.45/0.39	0.29/0.42	0.43/0.40	0.47/0.39	0.39/0.41	0.36/0.41	0.32/0.42
		**OR (95% CI)**	1.30 (0.87, 1.94)	0.50 (0.26, 0.95)	1.13 (0.71, 1.81)	1.39 (0.91, 2.14)	0.92 (0.47, 1.83)	1.29 (0.72, 2.32)	0.67 (0.40, 1.14)
		**p-value**	0.2	0.0	0.6	0.1	0.8	0.4	0.1
ALDH1L1	rs12638724	**MAF**[a]	0.50/0.43	0.48/0.44	0.38/0.46	0.45/0.45	0.47/0.45	0.48/0.44	0.38/0.46
		**OR (95% CI)**	1.40 (0.91, 2.14)	1.19 (0.66, 2.16)	0.68 (0.42, 1.13)	0.99 (0.63, 1.55)	1.09 (0.52, 2.27)	0.82 (0.45, 1.49)	0.60 (0.34, 1.07)
		**p-value**	0.1	0.6	0.1	1.0	0.8	0.5	0.1
ALDH1L1	rs13060596	**MAF**[a]	0.39/0.33	0.23/0.36	0.38/0.34	0.41/0.33	0.31/0.35	0.26/0.35	0.26/0.36
		**OR (95% CI)**	1.36 (0.91, 2.04)	0.51 (0.26, 0.99)	1.21 (0.76, 1.95)	1.43 (0.93, 2.20)	0.81 (0.39, 1.67)	1.65 (0.88, 3.12)	0.65 (0.37, 1.14)
		**p-value**	0.1	0.0	0.4	0.1	0.6	0.1	0.1
ALDH1L1	rs1868138	**MAF**[a]	0.21/0.26	0.34/0.24	0.21/0.26	0.29/0.24	0.19/0.25	0.24/0.25	0.28/0.25
		**OR (95% CI)**	0.69 (0.42, 1.12)	1.72 (0.92, 3.19)	0.70 (0.40, 1.24)	1.31 (0.81, 2.11)	0.80 (0.34, 1.90)	0.97 (0.50, 1.88)	1.32 (0.73, 2.39)
		**p-value**	0.1	0.1	0.2	0.3	0.6	0.9	0.4
ALDH1L1	rs2003334	**MAF**[a]	0.36/0.37	0.41/0.36	0.31/0.38	0.38/0.37	0.36/0.37	0.45/0.36	0.38/0.37
		**OR (95% CI)**	0.92 (0.61, 1.40)	1.18 (0.66, 2.12)	0.72 (0.44, 1.19)	0.99 (0.63, 1.53)	0.98 (0.48, 2.00)	0.63 (0.35, 1.13)	1.04 (0.61, 1.79)
		**p-value**	0.7	0.6	0.2	0.9	1.0	0.1	0.9

		**MAF[a]**	0.10/0.06	0.09/0.07	0.06/0.07	0.05/0.08	0.08/0.07	0.07/0.07	0.06/0.07
ALDH1L1	rs2044576	**OR (95% CI)**	1.51 (0.74, 3.05)	1.23 (0.45, 3.35)	0.65 (0.24, 1.74)	0.59 (0.23, 1.48)	1.51 (0.44, 5.24)	0.89 (0.30, 2.65)	0.99 (0.34, 2.93)
		**p-value**	0.3	0.7	0.4	0.3	0.5	0.8	1.0
		**MAF[a]**	0.48/0.44	0.48/0.46	0.40/0.47	0.46/0.46	0.47/0.46	0.50/0.45	0.39/0.47
ALDH1L1	rs2290053	**OR (95% CI)**	1.42 (0.92, 2.18)	1.14 (0.63, 2.08)	0.72 (0.44, 1.19)	0.98 (0.62, 1.53)	1.06 (0.51, 2.20)	0.77 (0.42, 1.40)	0.61 (0.35, 1.09)
		**p-value**	0.1	0.7	0.2	0.9	0.9	0.4	0.1
		**MAF[a]**	0.27/0.27	0.27/0.27	0.28/0.27	0.29/0.27	0.25/0.27	0.28/0.27	0.25/0.28
ALDH1L1	rs3772411	**OR (95% CI)**	1.05 (0.66, 1.68)	0.96 (0.49, 1.88)	1.09 (0.64, 1.86)	1.14 (0.69, 1.87)	0.78 (0.34, 1.80)	1.04 (0.54, 1.99)	0.78 (0.42, 1.44)
		**p-value**	0.8	0.9	0.8	0.6	0.6	0.9	0.4
		**MAF[a]**	0.34/0.29	0.27/0.31	0.27/0.31	0.30/0.30	0.25/0.31	0.31/0.30	0.33/0.30
ALDH1L1	rs3772426	**OR (95% CI)**	1.34 (0.86, 2.10)	0.80 (0.41, 1.57)	0.82 (0.48, 1.40)	1.03 (0.63, 1.68)	0.69 (0.30, 1.56)	0.96 (0.52, 1.80)	1.06 (0.59, 1.90)
		**p-value**	0.2	0.5	0.5	0.9	0.4	0.9	0.8
		**MAF[a]**	0.35/0.36	0.23/0.37	0.41/0.35	0.34/0.37	0.42/0.36	0.28/0.37	0.49/0.34
ALDH1L1	rs3816366	**OR (95% CI)**	0.94 (0.61, 1.45)	0.50 (0.25, 0.99)	1.37 (0.83, 2.24)	0.93 (0.58, 1.49)	1.28 (0.61, 2.67)	1.69 (0.89, 3.21)	1.91 (1.08, 3.38)
		**p-value**	0.8	0.0	0.2	0.8	0.5	0.1	0.0
		**MAF[a]**	0.44/0.36	0.41/0.38	0.39/0.38	0.36/0.39	0.33/0.38	0.34/0.39	0.33/0.39
ALDH1L1	rs4646715	**OR (95% CI)**	1.37 (0.90, 2.10)	1.15 (0.63, 2.11)	1.00 (0.61, 1.63)	0.84 (0.53, 1.33)	0.84 (0.39, 1.81)	1.16 (0.63, 2.13)	0.80 (0.45, 1.42)
		**p-value**	0.1	0.6	1.0	0.5	0.7	0.6	0.5
		**MAF[a]**	0.43/0.37	0.41/0.38	0.39/0.38	0.37/0.39	0.33/0.39	0.34/0.39	0.35/0.39
ALDH1L1	rs4646717	**OR (95% CI)**	1.29 (0.85, 1.97)	1.14 (0.63, 2.08)	0.99 (0.61, 1.61)	0.87 (0.55, 1.38)	0.84 (0.39, 1.78)	1.17 (0.64, 2.14)	0.86 (0.49, 1.51)
		**p-value**	0.2	0.7	1.0	0.6	0.6	0.6	0.6
		**MAF[a]**	0.43/0.43	0.27/0.45	0.47/0.42	0.45/0.43	0.44/0.43	0.34/0.44	0.54/0.41
ALDH1L1	rs4646739	**OR (95% CI)**	1.04 (0.68, 1.58)	0.42 (0.22, 0.82)	1.26 (0.78, 2.04)	1.15 (0.74, 1.78)	1.00 (0.48, 2.09)	1.70 (0.91, 3.18)	1.77 (1.01, 3.13)
		**p-value**	0.9	0.0	0.4	0.5	1.0	0.1	0.0
		**MAF[a]**	0.20/0.23	0.29/0.22	0.28/0.21	0.20/0.23	0.17/0.23	0.22/0.22	0.21/0.23
ALDH1L1	rs4646745	**OR (95% CI)**	0.88 (0.54, 1.46)	1.45 (0.76, 2.79)	1.51 (0.88, 2.60)	0.81 (0.47, 1.39)	0.62 (0.24, 1.58)	1.08 (0.54, 2.15)	0.90 (0.47, 1.73)
		**p-value**	0.6	0.3	0.1	0.4	0.3	0.8	0.8
		**MAF[a]**	0.23/0.24	0.23/0.24	0.19/0.25	0.25/0.24	0.25/0.24	0.36/0.23	0.21/0.24
ALDH1L1	rs4646755	**OR (95% CI)**	1.02 (0.63, 1.66)	0.93 (0.46, 1.86)	0.72 (0.40, 1.32)	1.04 (0.62, 1.75)	0.94 (0.41, 2.11)	0.45 (0.23, 0.86)	0.73 (0.38, 1.38)
		**p-value**	0.9	0.8	0.3	0.9	0.9	0.0	0.3
		**MAF[a]**	0.40/0.44	0.56/0.42	0.38/0.44	0.44/0.43	0.44/0.43	0.48/0.43	0.42/0.43
ALDH1L1	rs4646761	**OR (95% CI)**	0.78 (0.51, 1.19)	1.82 (0.99, 3.31)	0.69 (0.42, 1.14)	0.94 (0.60, 1.47)	1.22 (0.60, 2.47)	0.69 (0.39, 1.23)	1.05 (0.61, 1.80)
		**p-value**	0.3	0.1	0.1	0.8	0.6	0.2	0.9
		**MAF[a]**	0.49/0.49	0.59/0.49	0.43/0.51	0.51/0.49	0.47/0.50	0.40/0.48	0.39/0.51
ALDH1L1	rs6774437	**OR (95% CI)**	0.92 (0.61, 1.40)	0.63 (0.35, 1.15)	1.38 (0.85, 2.25)	0.94 (0.60, 1.45)	1.16 (0.56, 2.37)	1.74 (0.96, 3.16)	1.91 (1.08, 3.37)
		**p-value**	0.7	0.1	0.2	0.8	0.7	0.1	0.0
		**MAF[a]**	0.36/0.40	0.32/0.40	0.42/0.38	0.37/0.40	0.44/0.39	0.38/0.39	0.49/0.38
ALDH1L1	rs7639966	**OR (95% CI)**	0.83 (0.54, 1.29)	0.72 (0.38, 1.37)	1.29 (0.78, 2.13)	0.92 (0.58, 1.48)	1.16 (0.55, 2.44)	1.12 (0.61, 2.05)	1.44 (0.82, 2.52)
		**p-value**	0.4	0.3	0.3	0.7	0.7	0.7	0.2
		**MAF[a]**	0.17/0.20	0.25/0.19	0.22/0.19	0.20/0.20	0.17/0.20	0.17/0.20	0.21/0.20
ALDH1L1	rs9847790	**OR (95% CI)**	0.75 (0.44, 1.28)	1.58 (0.78, 3.20)	1.15 (0.64, 2.06)	1.00 (0.57, 1.74)	0.88 (0.34, 2.32)	1.15 (0.53, 2.46)	1.16 (0.59, 2.29)
		**p-value**	0.3	0.2	0.6	1.0	0.8	0.7	0.7
		**MAF[a]**	0.13/0.15	0.07/0.15	0.17/0.14	0.15/0.14	0.17/0.14	0.19/0.14	0.14/0.14
ALDH1L2	rs10459204	**OR (95% CI)**	0.89 (0.51, 1.58)	0.42 (0.15, 1.22)	1.29 (0.70, 2.38)	1.13 (0.63, 2.02)	1.09 (0.44, 2.71)	0.73 (0.36, 1.46)	0.85 (0.41, 1.76)
		**p-value**	0.7	0.1	0.4	0.7	0.8	0.4	0.7
ALDH1L2	rs10746009	**MAF[a]**	0.40/0.38	0.39/0.38	0.41/0.38	0.37/0.39	0.42/0.38	0.43/0.38	0.27/0.40

		**OR (95% CI)**	1.10 (0.72, 1.67)	1.01 (0.55, 1.86)	1.14 (0.70, 1.85)	0.86 (0.54, 1.37)	1.25 (0.61, 2.58)	0.74 (0.41, 1.33)	0.55 (0.30, 0.99)
		**p-value**	0.7	1.0	0.6	0.5	0.5	0.3	0.0
ALDH1L2	rs10778364	**MAF[a]**	0.43/0.44	0.39/0.44	0.40/0.45	0.47/0.43	0.33/0.45	0.41/0.44	0.56/0.42
		**OR (95% CI)**	1.01 (0.68, 1.51)	0.82 (0.45, 1.48)	0.88 (0.55, 1.42)	1.23 (0.80, 1.89)	0.56 (0.27, 1.14)	1.14 (0.64, 2.02)	1.55 (0.94, 2.57)
		**p-value**	1.0	0.5	0.6	0.4	0.1	0.7	0.1
ALDH1L2	rs10861327	**MAF[a]**	0.48/0.42	0.41/0.44	0.36/0.45	0.46/0.43	0.56/0.43	0.47/0.43	0.33/0.45
		**OR (95% CI)**	1.39 (0.94, 2.07)	0.87 (0.49, 1.53)	0.73 (0.45, 1.16)	1.10 (0.72, 1.68)	1.44 (0.74, 2.80)	0.90 (0.52, 1.57)	0.56 (0.33, 0.95)
		**p-value**	0.1	0.6	0.2	0.7	0.3	0.7	0.0
ALDH1L2	rs12372293	**MAF[a]**	0.11/0.10	0.11/0.10	0.04/0.11	0.12/0.10	0.17/0.10	0.10/0.10	0.10/0.10
		**OR (95% CI)**	1.18 (0.61, 2.27)	1.11 (0.44, 2.77)	0.39 (0.13, 1.13)	1.33 (0.67, 2.62)	1.64 (0.64, 4.20)	1.08 (0.42, 2.75)	0.80 (0.34, 1.91)
		**p-value**	0.6	0.8	0.1	0.4	0.3	0.9	0.6
ALDH1L2	rs1345094	**MAF[a]**	0.36/0.38	0.27/0.39	0.44/0.36	0.40/0.37	0.33/0.38	0.36/0.38	0.41/0.37
		**OR (95% CI)**	0.89 (0.60, 1.34)	0.55 (0.29, 1.03)	1.33 (0.85, 2.11)	1.08 (0.71, 1.66)	0.89 (0.44, 1.81)	1.01 (0.57, 1.78)	1.31 (0.79, 2.18)
		**p-value**	0.6	0.1	0.2	0.7	0.8	1.0	0.3
ALDH1L2	rs2374449	**MAF[a]**	0.14/0.18	0.11/0.18	0.23/0.16	0.17/0.17	0.14/0.17	0.12/0.18	0.26/0.16
		**OR (95% CI)**	0.79 (0.46, 1.37)	0.50 (0.20, 1.24)	1.71 (0.97, 2.99)	0.94 (0.53, 1.64)	0.72 (0.27, 1.89)	1.60 (0.68, 3.76)	1.96 (1.07, 3.58)
		**p-value**	0.4	0.1	0.1	0.8	0.5	0.3	0.0
ALDH1L2	rs7299166	**MAF[a]**	0.20/0.25	0.20/0.25	0.30/0.23	0.24/0.24	0.19/0.24	0.17/0.25	0.36/0.22
		**OR (95% CI)**	0.76 (0.48, 1.23)	0.72 (0.36, 1.44)	1.46 (0.89, 2.39)	0.97 (0.60, 1.57)	0.73 (0.32, 1.69)	1.56 (0.77, 3.17)	2.00 (1.17, 3.41)
		**p-value**	0.3	0.4	0.1	0.9	0.5	0.2	0.0
ALDH1L2	rs7954946	**MAF[a]**	0.43/0.48	0.41/0.47	0.56/0.45	0.47/0.47	0.39/0.47	0.43/0.47	0.54/0.46
		**OR (95% CI)**	0.78 (0.52, 1.16)	0.75 (0.42, 1.34)	1.44 (0.90, 2.28)	0.99 (0.65, 1.51)	0.81 (0.40, 1.61)	1.10 (0.63, 1.94)	1.62 (0.97, 2.70)
		**p-value**	0.2	0.3	0.1	1.0	0.5	0.7	0.1
ALDH2	rs10744777	**MAF[a]**	0.44/0.44	0.52/0.43	0.50/0.43	0.48/0.43	0.31/0.45	0.31/0.46	0.41/0.45
		**OR (95% CI)**	0.84 (0.53, 1.32)	1.51 (0.81, 2.82)	1.13 (0.67, 1.90)	1.23 (0.76, 1.97)	0.68 (0.31, 1.50)	2.00 (1.04, 3.86)	1.18 (0.66, 2.12)
		**p-value**	0.4	0.2	0.7	0.4	0.3	0.0	0.6
ALDH2	rs16941669	**MAF[a]**	0.10/0.08	0.14/0.08	0.07/0.09	0.07/0.09	0.08/0.09	0.09/0.09	0.07/0.09
		**OR (95% CI)**	1.26 (0.63, 2.53)	1.93 (0.81, 4.59)	0.74 (0.30, 1.85)	0.72 (0.32, 1.63)	0.85 (0.24, 2.99)	1.01 (0.36, 2.79)	0.65 (0.23, 1.81)
		**p-value**	0.5	0.1	0.5	0.4	0.8	1.0	0.4
CYP1A2	rs2470890	**MAF[a]**	0.49/0.47	0.57/0.47	0.47/0.48	0.46/0.48	0.42/0.48	0.45/0.48	0.43/0.49
		**OR (95% CI)**	1.04 (0.69, 1.56)	1.52 (0.86, 2.68)	0.80 (0.49, 1.28)	0.87 (0.57, 1.35)	1.00 (0.51, 1.98)	1.06 (0.61, 1.85)	1.05 (0.63, 1.77)
		**p-value**	0.9	0.2	0.3	0.5	1.0	0.8	0.8
CYP1A2	rs2472304	**MAF[a]**	0.50/0.47	0.57/0.47	0.47/0.48	0.46/0.48	0.42/0.48	0.46/0.48	0.43/0.49
		**OR (95% CI)**	1.00 (0.66, 1.50)	1.54 (0.86, 2.76)	0.80 (0.49, 1.29)	0.88 (0.57, 1.36)	1.00 (0.51, 1.98)	1.00 (0.57, 1.75)	1.05 (0.62, 1.76)
		**p-value**	1.0	0.1	0.4	0.6	1.0	1.0	0.9
CYP1A2	rs762551	**MAF[a]**	0.30/0.34	0.39/0.32	0.32/0.33	0.31/0.33	0.31/0.33	0.34/0.33	0.33/0.33
		**OR (95% CI)**	0.82 (0.53, 1.27)	1.38 (0.77, 2.47)	0.92 (0.56, 1.52)	0.89 (0.56, 1.41)	0.98 (0.47, 2.05)	0.88 (0.49, 1.59)	1.16 (0.67, 2.01)
		**p-value**	0.4	0.3	0.8	0.6	1.0	0.7	0.6
CYP2B6	rs7250601	**MAF[a]**	0.20/0.24	0.30/0.23	0.24/0.23	0.29/0.22	0.19/0.24	0.28/0.23	0.14/0.25
		**OR (95% CI)**	0.76 (0.47, 1.23)	1.57 (0.86, 2.87)	1.00 (0.59, 1.69)	1.50 (0.94, 2.40)	0.85 (0.37, 1.95)	0.83 (0.45, 1.51)	0.49 (0.24, 1.02)
		**p-value**	0.3	0.1	1.0	0.1	0.7	0.5	0.1
CYP2C8	rs10509681	**MAF[a]**	0.05/0.09	0.07/0.08	0.11/0.07	0.11/0.07	0.03/0.08	0.10/0.08	0.07/0.08
		**OR (95% CI)**	0.52 (0.22, 1.25)	0.93 (0.32, 2.69)	1.71 (0.81, 3.58)	1.59 (0.79, 3.18)	0.30 (0.04, 2.15)	0.71 (0.28, 1.78)	0.72 (0.28, 1.84)
		**p-value**	0.1	0.9	0.2	0.2	0.2	0.5	0.5
CYP2C8	rs1058932	**MAF[a]**	0.27/0.19	0.14/0.22	0.23/0.21	0.22/0.21	0.17/0.21	0.19/0.21	0.14/0.22

		**OR (95% CI)**	1.45 (0.91, 2.32)	0.58 (0.26, 1.28)	1.09 (0.63, 1.89)	1.07 (0.64, 1.78)	0.84 (0.33, 2.13)	1.08 (0.53, 2.18)	0.59 (0.27, 1.25)
		**p-value**	0.1	0.2	0.8	0.8	0.7	0.8	0.2
		**MAF[a]**	0.36/0.33	0.25/0.35	0.44/0.32	0.37/0.33	0.28/0.34	0.29/0.34	0.26/0.35
CYP2C8	rs11188156	**OR (95% CI)**	1.07 (0.71, 1.62)	0.62 (0.33, 1.18)	1.68 (1.05, 2.69)	1.15 (0.74, 1.79)	0.76 (0.35, 1.66)	1.20 (0.65, 2.20)	0.60 (0.32, 1.09)
		**p-value**	0.8	0.1	0.0	0.5	0.5	0.6	0.1
		**MAF[a]**	0.17/0.10	0.07/0.12	0.07/0.13	0.12/0.12	0.11/0.12	0.16/0.11	0.10/0.12
CYP2C8	rs11572126	**OR (95% CI)**	1.88 (1.08, 3.26)	0.53 (0.19, 1.52)	0.49 (0.20, 1.16)	0.92 (0.49, 1.76)	0.92 (0.32, 2.68)	0.70 (0.32, 1.49)	0.78 (0.33, 1.85)
		**p-value**	0.0	0.2	0.1	0.8	0.9	0.4	0.6
		**MAF[a]**	0.05/0.09	0.07/0.08	0.11/0.07	0.11/0.07	0.03/0.08	0.10/0.08	0.07/0.08
CYP2C8	rs11572174	**OR (95% CI)**	0.52 (0.22, 1.25)	0.93 (0.32, 2.69)	1.71 (0.81, 3.58)	1.59 (0.79, 3.18)	0.30 (0.04, 2.15)	0.71 (0.28, 1.78)	0.72 (0.28, 1.84)
		**p-value**	0.1	0.9	0.2	0.2	0.2	0.5	0.5
		**MAF[a]**	0.33/0.35	0.45/0.33	0.24/0.37	0.33/0.35	0.42/0.34	0.34/0.35	0.43/0.33
CYP2C8	rs1891071	**OR (95% CI)**	0.90 (0.58, 1.38)	1.58 (0.88, 2.85)	0.55 (0.32, 0.94)	0.85 (0.54, 1.35)	1.42 (0.69, 2.95)	0.96 (0.53, 1.75)	1.76 (1.01, 3.09)
		**p-value**	0.6	0.1	0.0	0.5	0.3	0.9	0.0
		**MAF[a]**	0.36/0.35	0.36/0.35	0.33/0.35	0.31/0.36	0.36/0.35	0.40/0.34	0.36/0.35
CYP2C8	rs1934952	**OR (95% CI)**	1.06 (0.70, 1.62)	1.11 (0.61, 2.01)	0.93 (0.57, 1.51)	0.87 (0.55, 1.38)	1.01 (0.48, 2.12)	0.87 (0.49, 1.54)	1.02 (0.58, 1.79)
		**p-value**	0.8	0.7	0.8	0.5	1.0	0.6	0.9
		**MAF[a]**	0.16/0.16	0.27/0.15	0.11/0.17	0.13/0.16	0.17/0.16	0.09/0.17	0.22/0.15
CYP2C8	rs1934956	**OR (95% CI)**	0.97 (0.57, 1.65)	2.14 (1.09, 4.17)	0.60 (0.30, 1.20)	0.74 (0.40, 1.36)	1.21 (0.47, 3.08)	1.97 (0.76, 5.09)	1.90 (0.99, 3.67)
		**p-value**	0.9	0.0	0.1	0.3	0.7	0.2	0.1
		**MAF[a]**	0.30/0.29	0.30/0.29	0.29/0.29	0.28/0.30	0.25/0.30	0.34/0.29	0.28/0.29
CYP2C8	rs2185571	**OR (95% CI)**	1.09 (0.69, 1.71)	1.12 (0.59, 2.11)	1.04 (0.62, 1.75)	0.99 (0.60, 1.61)	0.68 (0.30, 1.55)	0.85 (0.47, 1.56)	0.83 (0.45, 1.52)
		**p-value**	0.7	0.7	0.9	1.0	0.4	0.6	0.5
		**MAF[a]**	0.28/0.32	0.38/0.30	0.21/0.33	0.33/0.30	0.36/0.30	0.33/0.31	0.36/0.30
CYP2C8	rs2275622	**OR (95% CI)**	0.85 (0.55, 1.34)	1.34 (0.73, 2.44)	0.55 (0.31, 0.96)	1.07 (0.67, 1.71)	1.29 (0.61, 2.71)	0.87 (0.47, 1.58)	1.48 (0.84, 2.62)
		**p-value**	0.5	0.3	0.0	0.8	0.5	0.6	0.2
		**MAF[a]**	0.07/0.07	0.07/0.07	0.06/0.07	0.07/0.07	0.06/0.07	0.07/0.07	0.07/0.07
CYP2C9	rs1057910	**OR (95% CI)**	1.13 (0.51, 2.49)	1.09 (0.36, 3.28)	0.85 (0.31, 2.30)	1.17 (0.51, 2.68)	0.72 (0.16, 3.20)	1.08 (0.35, 3.32)	1.07 (0.39, 2.95)
		**p-value**	0.8	0.9	0.8	0.7	0.7	0.9	0.9
		**MAF[a]**	0.14/0.19	0.18/0.18	0.22/0.17	0.23/0.17	0.11/0.18	0.21/0.18	0.13/0.19
CYP2C9	rs1934963	**OR (95% CI)**	0.72 (0.41, 1.24)	1.04 (0.50, 2.16)	1.44 (0.82, 2.53)	1.58 (0.94, 2.64)	0.54 (0.19, 1.57)	0.81 (0.40, 1.62)	0.59 (0.28, 1.24)
		**p-value**	0.2	0.9	0.2	0.1	0.3	0.5	0.2
		**MAF[a]**	0.19/0.21	0.21/0.21	0.18/0.21	0.21/0.21	0.22/0.20	0.22/0.20	0.25/0.20
CYP2C9	rs1934967	**OR (95% CI)**	0.91 (0.55, 1.50)	1.04 (0.53, 2.05)	0.86 (0.47, 1.55)	0.95 (0.57, 1.61)	1.03 (0.45, 2.36)	0.85 (0.44, 1.65)	1.39 (0.75, 2.58)
		**p-value**	0.7	0.9	0.6	0.9	0.9	0.6	0.3
		**MAF[a]**	0.10/0.09	0.13/0.09	0.04/0.10	0.11/0.09	0.11/0.09	0.07/0.10	0.13/0.09
CYP2C9	rs1934968	**OR (95% CI)**	1.06 (0.55, 2.05)	1.25 (0.53, 2.93)	0.38 (0.13, 1.10)	1.10 (0.55, 2.18)	1.28 (0.43, 3.86)	1.50 (0.52, 4.31)	1.79 (0.80, 4.04)
		**p-value**	0.9	0.6	0.1	0.8	0.7	0.5	0.2
		**MAF[a]**	0.17/0.14	0.20/0.14	0.10/0.16	0.14/0.15	0.11/0.15	0.21/0.14	0.11/0.15
CYP2C9	rs2298037	**OR (95% CI)**	1.33 (0.79, 2.23)	1.30 (0.66, 2.58)	0.62 (0.30, 1.28)	0.89 (0.50, 1.59)	0.68 (0.24, 1.93)	0.64 (0.33, 1.26)	0.69 (0.31, 1.54)
		**p-value**	0.3	0.4	0.2	0.7	0.5	0.2	0.4
		**MAF[a]**	0.05/0.10	0.09/0.09	0.13/0.08	0.13/0.08	0.06/0.09	0.12/0.09	0.06/0.10
CYP2C9	rs4917636	**OR (95% CI)**	0.51 (0.22, 1.17)	1.01 (0.38, 2.70)	1.88 (0.93, 3.80)	1.64 (0.84, 3.18)	0.50 (0.12, 2.13)	0.68 (0.28, 1.64)	0.47 (0.16, 1.32)
		**p-value**	0.1	1.0	0.1	0.1	0.3	0.4	0.1
CYP2C9	rs4918758	**MAF[a]**	0.34/0.35	0.38/0.34	0.34/0.34	0.38/0.33	0.22/0.35	0.43/0.33	0.26/0.36

		**OR (95% CI)**	0.99 (0.66, 1.48)	1.14 (0.65, 2.00)	1.01 (0.63, 1.62)	1.21 (0.79, 1.86)	0.53 (0.24, 1.18)	0.65 (0.37, 1.13)	0.65 (0.37, 1.15)
		**p-value**	1.0	0.7	1.0	0.4	0.1	0.1	0.1
		**MAF[a]**	0.05/0.10	0.09/0.09	0.12/0.08	0.13/0.08	0.06/0.09	0.12/0.09	0.06/0.09
CYP2C9	rs9332100	**OR (95% CI)**	0.53 (0.23, 1.20)	1.04 (0.39, 2.78)	1.69 (0.82, 3.48)	1.68 (0.86, 3.27)	0.51 (0.12, 2.17)	0.66 (0.27, 1.58)	0.47 (0.17, 1.34)
		**p-value**	0.1	0.9	0.2	0.1	0.4	0.3	0.2
		**MAF[a]**	0.20/0.23	0.14/0.23	0.29/0.21	0.21/0.23	0.22/0.22	0.21/0.23	0.25/0.22
CYP2C9	rs9332174	**OR (95% CI)**	0.88 (0.54, 1.42)	0.58 (0.27, 1.26)	1.54 (0.92, 2.58)	1.02 (0.61, 1.71)	0.92 (0.40, 2.11)	1.24 (0.63, 2.42)	1.04 (0.57, 1.93)
		**p-value**	0.6	0.2	0.1	0.9	0.8	0.5	0.9
		**MAF[a]**	0.05/0.10	0.09/0.09	0.12/0.08	0.13/0.08	0.06/0.09	0.12/0.09	0.06/0.09
CYP2C9	rs9332216	**OR (95% CI)**	0.53 (0.23, 1.20)	1.04 (0.39, 2.78)	1.69 (0.82, 3.48)	1.68 (0.86, 3.27)	0.51 (0.12, 2.17)	0.66 (0.27, 1.58)	0.47 (0.17, 1.34)
		**p-value**	0.1	0.9	0.2	0.1	0.4	0.3	0.2
		**MAF[a]**	0.05/0.10	0.09/0.09	0.12/0.08	0.13/0.08	0.06/0.09	0.12/0.09	0.06/0.10
CYP2C9	rs9332242	**OR (95% CI)**	0.54 (0.24, 1.23)	1.04 (0.39, 2.76)	1.69 (0.82, 3.47)	1.67 (0.86, 3.25)	0.51 (0.12, 2.16)	0.66 (0.27, 1.59)	0.47 (0.17, 1.33)
		**p-value**	0.1	0.9	0.2	0.1	0.4	0.4	0.2
		**MAF[a]**	0.42/0.44	0.52/0.43	0.54/0.42	0.39/0.45	0.44/0.44	0.40/0.44	0.38/0.45
CYP2D6	rs1135840	**OR (95% CI)**	0.95 (0.65, 1.39)	1.42 (0.84, 2.42)	1.68 (1.08, 2.61)	0.81 (0.54, 1.22)	0.98 (0.51, 1.89)	1.16 (0.67, 1.98)	0.68 (0.40, 1.14)
		**p-value**	0.8	0.2	0.0	0.3	1.0	0.6	0.1
		**MAF[a]**	0.24/0.25	0.28/0.25	0.27/0.25	0.25/0.25	0.28/0.25	0.24/0.25	0.22/0.25
CYP2E1	rs2070673	**OR (95% CI)**	0.77 (0.46, 1.28)	1.26 (0.62, 2.55)	0.91 (0.51, 1.62)	0.93 (0.54, 1.61)	1.88 (0.81, 4.37)	0.88 (0.43, 1.80)	1.17 (0.59, 2.29)
		**p-value**	0.3	0.5	0.8	0.8	0.1	0.7	0.7
		**MAF[a]**	0.18/0.17	0.13/0.18	0.18/0.17	0.15/0.17	0.25/0.16	0.17/0.17	0.17/0.17
CYP2E1	rs2515641	**OR (95% CI)**	0.95 (0.56, 1.60)	0.70 (0.31, 1.61)	0.87 (0.47, 1.62)	0.83 (0.46, 1.48)	2.41 (1.09, 5.33)	0.88 (0.42, 1.87)	1.34 (0.67, 2.68)
		**p-value**	0.8	0.4	0.7	0.5	0.0	0.7	0.4
		**MAF[a]**	0.21/0.19	0.18/0.20	0.22/0.19	0.20/0.20	0.19/0.20	0.19/0.20	0.18/0.20
CYP3A4	rs12333983	**OR (95% CI)**	0.88 (0.50, 1.54)	0.85 (0.38, 1.94)	0.94 (0.49, 1.78)	0.87 (0.47, 1.60)	1.70 (0.66, 4.36)	0.83 (0.37, 1.82)	1.30 (0.62, 2.73)
		**p-value**	0.7	0.7	0.8	0.7	0.3	0.6	0.5
		**MAF[a]**	0.36/0.43	0.46/0.40	0.41/0.41	0.44/0.40	0.42/0.41	0.45/0.41	0.39/0.41
ERCC5	rs1047768	**OR (95% CI)**	0.75 (0.50, 1.13)	1.24 (0.71, 2.17)	1.02 (0.65, 1.62)	1.12 (0.73, 1.72)	1.00 (0.50, 2.02)	0.83 (0.48, 1.42)	0.88 (0.51, 1.51)
		**p-value**	0.2	0.4	0.9	0.6	1.0	0.5	0.6
		**MAF[a]**	0.28/0.26	0.27/0.26	0.32/0.25	0.26/0.26	0.14/0.27	0.24/0.26	0.24/0.27
ERCC5	rs17655	**OR (95% CI)**	1.05 (0.66, 1.67)	1.04 (0.54, 2.01)	1.34 (0.80, 2.25)	0.95 (0.57, 1.56)	0.49 (0.18, 1.33)	1.06 (0.55, 2.05)	0.97 (0.52, 1.83)
		**p-value**	0.8	0.9	0.3	0.8	0.2	0.9	0.9
		**MAF[a]**	0.18/0.25	0.25/0.23	0.24/0.23	0.25/0.23	0.28/0.23	0.24/0.23	0.22/0.23
ERCC5	rs2016073	**OR (95% CI)**	0.65 (0.39, 1.08)	1.07 (0.54, 2.10)	1.04 (0.60, 1.80)	1.11 (0.66, 1.84)	1.48 (0.65, 3.37)	0.92 (0.47, 1.80)	1.09 (0.57, 2.08)
		**p-value**	0.1	0.9	0.9	0.7	0.3	0.8	0.8
		**MAF[a]**	0.17/0.17	0.20/0.17	0.22/0.16	0.20/0.17	0.11/0.18	0.14/0.18	0.13/0.18
ERCC5	rs2094258	**OR (95% CI)**	0.98 (0.57, 1.67)	1.20 (0.57, 2.50)	1.45 (0.81, 2.59)	1.23 (0.70, 2.15)	0.59 (0.20, 1.75)	1.38 (0.62, 3.09)	0.62 (0.28, 1.37)
		**p-value**	0.9	0.6	0.2	0.5	0.3	0.4	0.2
		**MAF[a]**	0.17/0.26	0.23/0.24	0.23/0.24	0.25/0.23	0.33/0.23	0.28/0.23	0.25/0.23
ERCC5	rs3759500	**OR (95% CI)**	0.61 (0.37, 1.03)	0.93 (0.47, 1.84)	1.01 (0.58, 1.75)	1.08 (0.65, 1.78)	1.69 (0.78, 3.65)	0.77 (0.41, 1.47)	1.08 (0.58, 2.01)
		**p-value**	0.1	0.8	1.0	0.8	0.2	0.4	0.8
		**MAF[a]**	0.18/0.15	0.09/0.16	0.12/0.16	0.12/0.17	0.19/0.15	0.19/0.15	0.21/0.15
ERCC5	rs4150351	**OR (95% CI)**	1.42 (0.82, 2.48)	0.51 (0.19, 1.36)	0.76 (0.37, 1.56)	0.69 (0.36, 1.34)	1.15 (0.46, 2.84)	0.79 (0.37, 1.69)	1.24 (0.63, 2.44)
		**p-value**	0.2	0.2	0.5	0.3	0.8	0.5	0.5
ERCC5	rs4150383	**MAF[a]**	0.11/0.18	0.16/0.17	0.12/0.18	0.19/0.16	0.25/0.16	0.24/0.16	0.18/0.16

		**OR (95% CI)**	0.57 (0.30, 1.07)	0.90 (0.40, 2.03)	0.69 (0.34, 1.41)	1.17 (0.65, 2.09)	1.66 (0.70, 3.92)	0.51 (0.25, 1.03)	1.05 (0.52, 2.13)
		**p-value**	0.1	0.8	0.3	0.6	0.2	0.1	0.9
ERCC5	rs4150386	**MAF[a]**	0.10/0.10	0.09/0.10	0.10/0.10	0.10/0.10	0.06/0.10	0.12/0.10	0.11/0.10
		**OR (95% CI)**	1.07 (0.54, 2.12)	0.89 (0.33, 2.39)	1.14 (0.52, 2.49)	1.00 (0.49, 2.04)	0.43 (0.10, 1.90)	0.75 (0.30, 1.85)	1.04 (0.45, 2.41)
		**p-value**	0.8	0.8	0.7	1.0	0.3	0.5	0.9
ERCC5	rs4743	**MAF[a]**	0.42/0.40	0.43/0.40	0.33/0.42	0.41/0.41	0.47/0.40	0.40/0.41	0.44/0.40
		**OR (95% CI)**	1.12 (0.75, 1.67)	1.14 (0.65, 2.01)	0.75 (0.46, 1.20)	1.06 (0.69, 1.63)	1.16 (0.59, 2.29)	1.09 (0.62, 1.90)	1.07 (0.64, 1.78)
		**p-value**	0.6	0.6	0.2	0.8	0.7	0.8	0.8
G6PC	rs161630	**MAF[a]**	0.13/0.10	0.14/0.10	0.09/0.11	0.09/0.11	0.14/0.11	0.09/0.11	0.08/0.11
		**OR (95% CI)**	1.28 (0.68, 2.39)	1.54 (0.67, 3.59)	0.70 (0.31, 1.59)	0.80 (0.38, 1.68)	1.57 (0.57, 4.36)	1.39 (0.52, 3.73)	0.75 (0.29, 1.94)
		**p-value**	0.4	0.3	0.4	0.6	0.4	0.5	0.6
G6PC	rs2229611	**MAF[a]**	0.30/0.25	0.36/0.25	0.22/0.26	0.20/0.27	0.22/0.26	0.29/0.25	0.24/0.26
		**OR (95% CI)**	1.25 (0.80, 1.96)	1.68 (0.93, 3.06)	0.73 (0.42, 1.27)	0.64 (0.38, 1.08)	0.89 (0.39, 2.04)	0.84 (0.45, 1.58)	1.01 (0.55, 1.86)
		**p-value**	0.3	0.1	0.3	0.1	0.8	0.6	1.0
G6PC	rs324079	**MAF[a]**	0.16/0.14	0.18/0.14	0.13/0.15	0.11/0.15	0.08/0.15	0.21/0.14	0.15/0.14
		**OR (95% CI)**	1.13 (0.68, 1.88)	1.21 (0.61, 2.40)	0.88 (0.47, 1.64)	0.66 (0.36, 1.23)	0.57 (0.18, 1.83)	0.63 (0.32, 1.23)	1.21 (0.62, 2.37)
		**p-value**	0.6	0.6	0.7	0.2	0.3	0.2	0.6
GSTP1	rs1138272	**MAF[a]**	0.08/0.08	0.11/0.08	0.07/0.09	0.05/0.09	0.14/0.08	0.09/0.08	0.11/0.08
		**OR (95% CI)**	0.94 (0.45, 1.97)	1.44 (0.57, 3.65)	0.85 (0.34, 2.09)	0.57 (0.23, 1.39)	1.65 (0.60, 4.55)	0.91 (0.33, 2.49)	1.20 (0.52, 2.79)
		**p-value**	0.9	0.4	0.7	0.2	0.3	0.8	0.7
GSTP1	rs1695	**MAF[a]**	0.36/0.35	0.34/0.36	0.33/0.36	0.32/0.36	0.44/0.35	0.43/0.35	0.35/0.36
		**OR (95% CI)**	1.01 (0.68, 1.51)	0.93 (0.52, 1.64)	0.91 (0.57, 1.45)	0.82 (0.53, 1.28)	1.43 (0.73, 2.79)	0.70 (0.41, 1.19)	0.90 (0.53, 1.53)
		**p-value**	0.9	0.8	0.7	0.4	0.3	0.2	0.7
GSTP1	rs6591256	**MAF[a]**	0.43/0.39	0.38/0.41	0.36/0.41	0.35/0.42	0.50/0.40	0.47/0.40	0.43/0.40
		**OR (95% CI)**	1.20 (0.81, 1.79)	0.89 (0.50, 1.58)	0.83 (0.52, 1.34)	0.74 (0.48, 1.15)	1.41 (0.72, 2.79)	0.73 (0.42, 1.27)	1.03 (0.61, 1.72)
		**p-value**	0.4	0.7	0.4	0.2	0.3	0.3	0.9
GSTT1	deletion	**MAF[a]**	0.15/0.22	0.25/0.20	0.09/0.23	0.27/0.19	0.17/0.21	0.34/0.19	0.19/0.21
		**OR (95% CI)**	1.62 (0.76, 3.44)	0.74 (0.30, 1.87)	3.13 (1.06, 9.21)	0.65 (0.32, 1.30)	1.27 (0.35, 4.59)	2.66 (1.13, 6.28)	1.10 (0.44, 2.75)
		**p-value**	0.2	0.5	0.0	0.2	0.7	0.0	0.8
HIF1A	rs10873142	**MAF[a]**	0.27/0.22	0.29/0.23	0.22/0.24	0.25/0.23	0.17/0.24	0.16/0.24	0.22/0.24
		**OR (95% CI)**	1.16 (0.71, 1.89)	1.37 (0.70, 2.66)	0.72 (0.40, 1.32)	1.06 (0.63, 1.79)	0.90 (0.36, 2.29)	1.76 (0.79, 3.93)	1.36 (0.72, 2.59)
		**p-value**	0.5	0.4	0.3	0.8	0.8	0.2	0.3
HIF1A	rs11549465	**MAF[a]**	0.11/0.07	0.05/0.08	0.10/0.08	0.06/0.09	0.03/0.08	0.09/0.08	0.08/0.08
		**OR (95% CI)**	1.54 (0.81, 2.93)	0.59 (0.18, 1.93)	1.32 (0.63, 2.78)	0.74 (0.33, 1.68)	0.32 (0.04, 2.31)	1.06 (0.41, 2.76)	1.14 (0.48, 2.73)
		**p-value**	0.2	0.4	0.5	0.5	0.3	0.9	0.8
HIF1A	rs12434438	**MAF[a]**	0.33/0.27	0.30/0.28	0.31/0.28	0.28/0.28	0.17/0.29	0.22/0.29	0.25/0.29
		**OR (95% CI)**	1.16 (0.73, 1.86)	1.13 (0.58, 2.18)	0.96 (0.56, 1.66)	0.90 (0.54, 1.51)	0.70 (0.27, 1.80)	1.34 (0.66, 2.71)	1.24 (0.66, 2.32)
		**p-value**	0.5	0.7	0.9	0.7	0.5	0.4	0.5
HIF1A	rs2057482	**MAF[a]**	0.20/0.15	0.18/0.16	0.17/0.16	0.17/0.16	0.11/0.17	0.12/0.17	0.15/0.17
		**OR (95% CI)**	1.27 (0.75, 2.13)	1.01 (0.48, 2.16)	0.89 (0.47, 1.66)	0.98 (0.55, 1.74)	0.76 (0.26, 2.26)	1.56 (0.66, 3.68)	1.27 (0.62, 2.59)
		**p-value**	0.4	1.0	0.7	0.9	0.6	0.3	0.5
HIF1A	rs2301113	**MAF[a]**	0.34/0.30	0.34/0.31	0.37/0.30	0.30/0.31	0.25/0.31	0.21/0.32	0.29/0.31
		**OR (95% CI)**	1.03 (0.67, 1.61)	1.19 (0.64, 2.22)	1.13 (0.68, 1.87)	0.93 (0.58, 1.51)	1.04 (0.48, 2.25)	1.80 (0.89, 3.65)	1.23 (0.70, 2.17)
		**p-value**	0.9	0.6	0.6	0.8	0.9	0.1	0.5
HNF4A	rs11086926	**MAF[a]**	0.11/0.11	0.13/0.11	0.16/0.10	0.10/0.12	0.00/0.12	0.14/0.11	0.11/0.11

		**OR (95% CI)**	0.94 (0.50, 1.75)	1.18 (0.50, 2.79)	1.45 (0.75, 2.80)	0.83 (0.41, 1.67)	0.00 (0.00, 0.00)	0.76 (0.34, 1.74)	1.14 (0.50, 2.59)
		**p-value**	0.8	0.7	0.3	0.6	0.9	0.5	0.8
HNF4A	rs11574736	**MAF[a]**	0.15/0.18	0.14/0.17	0.14/0.18	0.18/0.17	0.25/0.16	0.22/0.16	0.17/0.17
		**OR (95% CI)**	0.88 (0.50, 1.54)	0.79 (0.35, 1.79)	0.85 (0.44, 1.66)	1.09 (0.62, 1.94)	1.56 (0.67, 3.63)	0.64 (0.31, 1.32)	0.87 (0.42, 1.78)
		**p-value**	0.6	0.6	0.6	0.8	0.3	0.2	0.7
HNF4A	rs2071200	**MAF[a]**	0.30/0.33	0.32/0.32	0.37/0.31	0.32/0.32	0.36/0.32	0.29/0.32	0.31/0.32
		**OR (95% CI)**	0.79 (0.51, 1.24)	1.04 (0.56, 1.93)	1.24 (0.75, 2.04)	0.98 (0.61, 1.56)	1.48 (0.68, 3.19)	1.05 (0.56, 1.97)	1.02 (0.56, 1.86)
		**p-value**	0.3	0.9	0.4	0.9	0.3	0.9	0.9
HNF4A	rs2425640	**MAF[a]**	0.39/0.36	0.43/0.36	0.37/0.37	0.32/0.38	0.39/0.36	0.36/0.37	0.33/0.37
		**OR (95% CI)**	1.13 (0.74, 1.72)	1.43 (0.80, 2.58)	1.01 (0.62, 1.65)	0.78 (0.49, 1.24)	1.12 (0.53, 2.35)	1.00 (0.55, 1.84)	0.79 (0.44, 1.41)
		**p-value**	0.6	0.2	1.0	0.3	0.8	1.0	0.4
HNF4A	rs3212199	**MAF[a]**	0.46/0.41	0.39/0.43	0.39/0.43	0.39/0.43	0.50/0.42	0.41/0.43	0.44/0.42
		**OR (95% CI)**	1.18 (0.79, 1.76)	0.86 (0.48, 1.53)	0.79 (0.49, 1.27)	0.80 (0.52, 1.24)	1.59 (0.78, 3.23)	0.98 (0.55, 1.73)	1.28 (0.74, 2.19)
		**p-value**	0.4	0.6	0.3	0.3	0.2	0.9	0.4
HNF4A	rs3818247	**MAF[a]**	0.43/0.38	0.41/0.39	0.39/0.39	0.34/0.41	0.39/0.39	0.38/0.39	0.40/0.39
		**OR (95% CI)**	1.22 (0.80, 1.85)	1.08 (0.60, 1.95)	0.92 (0.56, 1.49)	0.72 (0.46, 1.14)	1.09 (0.52, 2.25)	1.08 (0.59, 1.98)	1.22 (0.71, 2.12)
		**p-value**	0.4	0.8	0.7	0.2	0.8	0.8	0.5
HNF4A	rs6031595	**MAF[a]**	0.44/0.47	0.46/0.46	0.42/0.47	0.54/0.44	0.50/0.46	0.45/0.46	0.43/0.47
		**OR (95% CI)**	0.96 (0.64, 1.44)	0.99 (0.56, 1.76)	0.90 (0.56, 1.45)	1.55 (1.00, 2.42)	0.99 (0.50, 1.95)	1.14 (0.65, 2.00)	0.70 (0.42, 1.19)
		**p-value**	0.8	1.0	0.7	0.1	1.0	0.6	0.2
HNF4A	rs6073432	**MAF[a]**	0.33/0.31	0.32/0.31	0.27/0.32	0.35/0.31	0.39/0.31	0.29/0.32	0.26/0.32
		**OR (95% CI)**	1.12 (0.73, 1.72)	1.01 (0.54, 1.88)	0.72 (0.42, 1.21)	1.25 (0.79, 1.98)	1.47 (0.72, 2.99)	1.20 (0.65, 2.23)	0.77 (0.43, 1.36)
		**p-value**	0.6	1.0	0.2	0.3	0.3	0.6	0.4
HNF4A	rs6093978	**MAF[a]**	0.37/0.32	0.32/0.34	0.29/0.34	0.37/0.33	0.39/0.33	0.33/0.34	0.26/0.35
		**OR (95% CI)**	1.28 (0.84, 1.95)	0.92 (0.49, 1.70)	0.76 (0.45, 1.26)	1.25 (0.79, 1.97)	1.27 (0.62, 2.61)	1.13 (0.61, 2.07)	0.66 (0.37, 1.18)
		**p-value**	0.3	0.8	0.3	0.3	0.5	0.7	0.2
HNF4A	rs6130615	**MAF[a]**	0.20/0.19	0.20/0.19	0.23/0.18	0.19/0.19	0.19/0.19	0.21/0.19	0.13/0.20
		**OR (95% CI)**	0.85 (0.47, 1.52)	1.04 (0.45, 2.40)	1.15 (0.60, 2.21)	0.87 (0.46, 1.64)	1.72 (0.63, 4.67)	0.72 (0.32, 1.62)	0.75 (0.32, 1.72)
		**p-value**	0.6	0.9	0.7	0.7	0.3	0.4	0.5
HNF4A	rs8114057	**MAF[a]**	0.48/0.49	0.50/0.50	0.56/0.49	0.40/0.52	0.58/0.49	0.48/0.50	0.50/0.50
		**OR (95% CI)**	1.10 (0.74, 1.64)	1.02 (0.58, 1.78)	1.32 (0.84, 2.09)	0.61 (0.40, 0.95)	1.52 (0.74, 3.11)	1.10 (0.63, 1.91)	1.04 (0.61, 1.77)
		**p-value**	0.6	0.9	0.2	0.0	0.3	0.7	0.9
MDM2	rs1625525	**MAF[a]**	0.32/0.39	0.36/0.38	0.39/0.37	0.40/0.37	0.44/0.37	0.38/0.38	0.42/0.37
		**OR (95% CI)**	0.76 (0.50, 1.15)	0.92 (0.52, 1.63)	1.14 (0.71, 1.82)	1.21 (0.79, 1.87)	1.19 (0.61, 2.35)	1.09 (0.62, 1.90)	1.10 (0.65, 1.85)
		**p-value**	0.2	0.8	0.6	0.4	0.6	0.8	0.7
MDM2	rs1846402	**MAF[a]**	0.13/0.17	0.20/0.16	0.13/0.17	0.18/0.16	0.25/0.16	0.21/0.16	0.13/0.17
		**OR (95% CI)**	0.75 (0.42, 1.32)	1.23 (0.60, 2.51)	0.81 (0.42, 1.58)	1.10 (0.63, 1.92)	1.71 (0.76, 3.80)	0.67 (0.33, 1.35)	0.68 (0.32, 1.45)
		**p-value**	0.3	0.6	0.5	0.7	0.2	0.3	0.3
MDM2	rs3730581	**MAF[a]**	0.40/0.33	0.30/0.35	0.24/0.37	0.38/0.34	0.28/0.35	0.38/0.35	0.39/0.34
		**OR (95% CI)**	1.40 (0.94, 2.09)	0.80 (0.44, 1.45)	0.59 (0.35, 0.98)	1.16 (0.76, 1.78)	0.64 (0.30, 1.34)	0.88 (0.50, 1.54)	1.10 (0.66, 1.84)
		**p-value**	0.1	0.5	0.0	0.5	0.2	0.6	0.7
NAT2	rs11780272	**MAF[a]**	0.42/0.39	0.36/0.41	0.37/0.41	0.38/0.41	0.33/0.41	0.48/0.39	0.42/0.40
		**OR (95% CI)**	1.16 (0.79, 1.71)	0.83 (0.48, 1.46)	0.88 (0.56, 1.39)	0.95 (0.63, 1.44)	0.69 (0.35, 1.39)	0.73 (0.43, 1.26)	0.99 (0.60, 1.63)
		**p-value**	0.4	0.5	0.6	0.8	0.3	0.3	1.0
NAT2	rs1208	**MAF[a]**	0.43/0.41	0.39/0.41	0.40/0.41	0.39/0.42	0.33/0.42	0.48/0.40	0.40/0.41

		**OR (95% CI)**	1.10 (0.76, 1.61)	0.93 (0.54, 1.60)	0.95 (0.61, 1.47)	0.93 (0.62, 1.39)	0.71 (0.36, 1.42)	0.75 (0.44, 1.29)	0.96 (0.59, 1.57)
		**p-value**	0.6	0.8	0.8	0.7	0.3	0.3	0.9
		**MAF[a]**	0.33/0.32	0.30/0.32	0.26/0.33	0.28/0.33	0.50/0.31	0.28/0.33	0.43/0.30
NAT2	rs17516895	**OR (95% CI)**	1.07 (0.71, 1.63)	0.91 (0.50, 1.67)	0.71 (0.43, 1.19)	0.79 (0.49, 1.25)	2.14 (1.06, 4.32)	1.35 (0.73, 2.49)	1.76 (1.03, 3.02)
		**p-value**	0.7	0.8	0.2	0.3	0.0	0.3	0.0
		**MAF[a]**	0.44/0.42	0.38/0.43	0.41/0.43	0.41/0.43	0.39/0.43	0.48/0.42	0.42/0.42
NAT2	rs1801280	**OR (95% CI)**	1.11 (0.76, 1.62)	0.82 (0.47, 1.42)	0.97 (0.62, 1.51)	0.97 (0.65, 1.46)	0.83 (0.43, 1.61)	0.80 (0.47, 1.38)	0.92 (0.56, 1.50)
		**p-value**	0.6	0.5	0.9	0.9	0.6	0.4	0.7
		**MAF[a]**	0.30/0.32	0.34/0.31	0.40/0.30	0.36/0.30	0.22/0.32	0.29/0.32	0.24/0.33
NAT2	rs1961456	**OR (95% CI)**	0.86 (0.55, 1.35)	1.14 (0.61, 2.13)	1.62 (0.99, 2.67)	1.27 (0.80, 2.02)	0.58 (0.25, 1.35)	1.09 (0.58, 2.05)	0.59 (0.32, 1.11)
		**p-value**	0.5	0.7	0.1	0.3	0.2	0.8	0.1
		**MAF[a]**	0.27/0.28	0.30/0.28	0.18/0.30	0.27/0.28	0.44/0.27	0.24/0.28	0.39/0.26
NAT2	rs4646242	**OR (95% CI)**	0.93 (0.60, 1.43)	1.13 (0.62, 2.05)	0.52 (0.30, 0.93)	0.94 (0.59, 1.49)	2.09 (1.05, 4.17)	1.26 (0.68, 2.34)	1.80 (1.05, 3.08)
		**p-value**	0.7	0.7	0.0	0.8	0.0	0.5	0.0
		**MAF[a]**	0.13/0.17	0.16/0.16	0.17/0.16	0.21/0.15	0.08/0.16	0.22/0.15	0.11/0.17
NAT2	rs4646243	**OR (95% CI)**	0.71 (0.39, 1.29)	0.95 (0.43, 2.13)	1.06 (0.56, 2.02)	1.64 (0.93, 2.87)	0.44 (0.13, 1.53)	0.57 (0.28, 1.19)	0.67 (0.30, 1.49)
		**p-value**	0.3	0.9	0.9	0.1	0.2	0.1	0.3
		**MAF[a]**	0.18/0.21	0.21/0.21	0.23/0.20	0.24/0.20	0.17/0.21	0.24/0.20	0.15/0.21
NAT2	rs4646246	**OR (95% CI)**	0.77 (0.46, 1.31)	1.04 (0.50, 2.15)	1.18 (0.66, 2.09)	1.31 (0.77, 2.22)	0.80 (0.31, 2.08)	0.75 (0.37, 1.51)	0.71 (0.34, 1.45)
		**p-value**	0.3	0.9	0.6	0.3	0.7	0.4	0.3
		**MAF[a]**	0.27/0.30	0.30/0.29	0.21/0.31	0.28/0.29	0.47/0.28	0.26/0.29	0.39/0.28
NAT2	rs6984200	**OR (95% CI)**	0.86 (0.56, 1.33)	1.07 (0.59, 1.93)	0.62 (0.36, 1.06)	0.93 (0.59, 1.47)	2.28 (1.14, 4.55)	1.21 (0.67, 2.21)	1.73 (1.01, 2.95)
		**p-value**	0.5	0.8	0.1	0.8	0.0	0.5	0.0
		**MAF[a]**	0.45/0.41	0.38/0.42	0.42/0.41	0.39/0.42	0.36/0.42	0.48/0.41	0.38/0.42
NAT2	rs7832071	**OR (95% CI)**	1.15 (0.80, 1.67)	0.86 (0.50, 1.47)	1.02 (0.67, 1.57)	0.92 (0.62, 1.37)	0.80 (0.41, 1.57)	0.78 (0.46, 1.32)	0.84 (0.51, 1.37)
		**p-value**	0.4	0.6	0.9	0.7	0.5	0.3	0.5
		**MAF[a]**	0.13/0.12	0.13/0.12	0.19/0.11	0.11/0.13	0.08/0.13	0.14/0.12	0.07/0.13
NFE2L2	rs13401026	**OR (95% CI)**	1.00 (0.56, 1.81)	1.03 (0.44, 2.39)	1.70 (0.92, 3.15)	0.80 (0.41, 1.56)	0.74 (0.22, 2.54)	0.85 (0.38, 1.89)	0.56 (0.21, 1.48)
		**p-value**	1.0	0.9	0.1	0.5	0.6	0.7	0.2
		**MAF[a]**	0.11/0.08	0.07/0.08	0.08/0.08	0.06/0.09	0.08/0.08	0.07/0.08	0.08/0.08
NFE2L2	rs2588882	**OR (95% CI)**	1.44 (0.75, 2.75)	0.84 (0.29, 2.41)	0.95 (0.42, 2.17)	0.68 (0.30, 1.55)	1.00 (0.30, 3.35)	1.15 (0.40, 3.32)	0.96 (0.39, 2.40)
		**p-value**	0.3	0.7	0.9	0.4	1.0	0.8	0.9
		**MAF[a]**	0.27/0.24	0.25/0.25	0.31/0.23	0.21/0.25	0.22/0.25	0.22/0.25	0.18/0.26
NFE2L2	rs8470	**OR (95% CI)**	1.09 (0.70, 1.70)	1.05 (0.55, 1.98)	1.32 (0.80, 2.17)	0.80 (0.48, 1.31)	1.08 (0.47, 2.49)	1.08 (0.56, 2.08)	0.75 (0.39, 1.45)
		**p-value**	0.7	0.9	0.3	0.4	0.9	0.8	0.4
		**MAF[a]**	0.28/0.24	0.25/0.25	0.32/0.24	0.21/0.26	0.22/0.25	0.24/0.25	0.19/0.26
NFE2L2	rs9068	**OR (95% CI)**	1.08 (0.70, 1.69)	1.01 (0.53, 1.91)	1.34 (0.81, 2.20)	0.75 (0.45, 1.23)	1.07 (0.46, 2.46)	0.99 (0.52, 1.89)	0.81 (0.42, 1.55)
		**p-value**	0.7	1.0	0.3	0.3	0.9	1.0	0.5
		**MAF[a]**	0.34/0.38	0.43/0.36	0.34/0.37	0.41/0.36	0.50/0.36	0.28/0.38	0.35/0.37
NOS2A	rs10459953	**OR (95% CI)**	0.87 (0.59, 1.28)	1.34 (0.79, 2.27)	0.91 (0.58, 1.43)	1.25 (0.83, 1.86)	1.66 (0.86, 3.21)	1.56 (0.86, 2.84)	0.83 (0.49, 1.39)
		**p-value**	0.5	0.3	0.7	0.3	0.1	0.1	0.5
		**MAF[a]**	0.27/0.19	0.16/0.22	0.21/0.21	0.19/0.22	0.11/0.22	0.29/0.20	0.18/0.22
NOS2A	rs1113283	**OR (95% CI)**	1.47 (0.96, 2.26)	0.72 (0.35, 1.45)	0.99 (0.59, 1.67)	0.83 (0.50, 1.37)	0.47 (0.17, 1.31)	0.64 (0.36, 1.14)	0.78 (0.41, 1.47)
		**p-value**	0.1	0.4	1.0	0.5	0.1	0.1	0.4
NOS2A	rs2297516	**MAF[a]**	0.39/0.41	0.46/0.40	0.44/0.40	0.35/0.42	0.47/0.40	0.36/0.41	0.44/0.40
		**OR (95% CI)**	0.94 (0.62, 1.40)	1.26 (0.72, 2.21)	1.20 (0.75, 1.91)	0.72 (0.46, 1.11)	1.34 (0.68, 2.68)	1.27 (0.71, 2.27)	1.24 (0.73, 2.10)

		**p-value**	0.7	0.4	0.4	0.1	0.4	0.4	0.4
NOS2A	rs2297518	**MAF[a]**	0.18/0.16	0.13/0.17	0.12/0.17	0.15/0.17	0.14/0.17	0.26/0.15	0.18/0.16
		**OR (95% CI)**	1.16 (0.69, 1.94)	0.71 (0.32, 1.61)	0.71 (0.36, 1.38)	0.91 (0.52, 1.60)	0.76 (0.29, 2.01)	0.53 (0.28, 1.00)	1.07 (0.55, 2.09)
		**p-value**	0.6	0.4	0.3	0.7	0.6	0.1	0.8
NOS2A	rs2779252	**MAF[a]**	0.05/0.06	0.04/0.06	0.07/0.06	0.08/0.05	0.00/0.06	0.07/0.06	0.06/0.06
		**OR (95% CI)**	0.87 (0.39, 1.93)	0.60 (0.15, 2.39)	1.10 (0.47, 2.55)	1.42 (0.68, 2.98)	0.00 (0.00, 0.00)	0.80 (0.29, 2.21)	1.23 (0.45, 3.36)
		**p-value**	0.7	0.5	0.8	0.3	1.0	0.7	0.7
NOS2A	rs3794763	**MAF[a]**	0.22/0.23	0.21/0.23	0.34/0.21	0.19/0.24	0.19/0.23	0.16/0.24	0.25/0.22
		**OR (95% CI)**	0.93 (0.58, 1.51)	0.85 (0.43, 1.69)	2.05 (1.22, 3.43)	0.71 (0.42, 1.22)	0.86 (0.37, 2.01)	1.81 (0.85, 3.87)	1.35 (0.74, 2.46)
		**p-value**	0.8	0.6	0.0	0.2	0.7	0.1	0.3
NOS2A	rs4795067	**MAF[a]**	0.37/0.31	0.25/0.33	0.28/0.34	0.29/0.34	0.28/0.33	0.47/0.31	0.33/0.33
		**OR (95% CI)**	1.29 (0.87, 1.92)	0.68 (0.37, 1.24)	0.79 (0.49, 1.27)	0.80 (0.51, 1.24)	0.77 (0.37, 1.62)	0.55 (0.32, 0.93)	1.03 (0.61, 1.76)
		**p-value**	0.2	0.2	0.3	0.3	0.5	0.0	0.9
NOS2A	rs4796052	**MAF[a]**	0.19/0.18	0.13/0.19	0.16/0.19	0.19/0.18	0.14/0.19	0.28/0.17	0.19/0.18
		**OR (95% CI)**	1.11 (0.67, 1.81)	0.62 (0.27, 1.40)	0.82 (0.45, 1.51)	1.06 (0.62, 1.79)	0.67 (0.25, 1.75)	0.57 (0.31, 1.06)	1.01 (0.53, 1.92)
		**p-value**	0.7	0.2	0.5	0.8	0.4	0.1	1.0
NOS2A	rs8072199	**MAF[a]**	0.45/0.37	0.30/0.40	0.36/0.40	0.34/0.40	0.56/0.38	0.38/0.39	0.38/0.39
		**OR (95% CI)**	1.54 (1.02, 2.31)	0.68 (0.37, 1.24)	0.92 (0.57, 1.48)	0.78 (0.50, 1.22)	1.82 (0.91, 3.62)	1.06 (0.60, 1.88)	0.78 (0.46, 1.33)
		**p-value**	0.0	0.2	0.7	0.3	0.1	0.8	0.4
NOS2A	rs944725	**MAF[a]**	0.43/0.37	0.29/0.40	0.49/0.37	0.30/0.41	0.31/0.39	0.45/0.38	0.39/0.39
		**OR (95% CI)**	1.27 (0.84, 1.91)	0.57 (0.30, 1.06)	1.64 (1.02, 2.63)	0.60 (0.38, 0.95)	0.71 (0.34, 1.50)	0.74 (0.42, 1.30)	1.10 (0.64, 1.88)
		**p-value**	0.3	0.1	0.0	0.0	0.4	0.3	0.7
NQO1	rs10517	**MAF[a]**	0.16/0.17	0.13/0.17	0.26/0.15	0.18/0.16	0.19/0.16	0.17/0.17	0.07/0.18
		**OR (95% CI)**	0.92 (0.55, 1.56)	0.66 (0.29, 1.51)	1.91 (1.11, 3.29)	1.05 (0.61, 1.81)	1.29 (0.55, 3.06)	0.92 (0.45, 1.89)	0.37 (0.14, 0.97)
		**p-value**	0.8	0.3	0.0	0.9	0.6	0.8	0.0
NQO1	rs17298718	**MAF[a]**	0.06/0.07	0.11/0.07	0.09/0.07	0.04/0.08	0.06/0.07	0.07/0.07	0.08/0.07
		**OR (95% CI)**	0.84 (0.38, 1.87)	1.71 (0.69, 4.21)	1.41 (0.63, 3.15)	0.58 (0.22, 1.52)	0.72 (0.17, 3.10)	1.00 (0.35, 2.83)	1.07 (0.41, 2.79)
		**p-value**	0.7	0.2	0.4	0.3	0.7	1.0	0.9
NQO1	rs1800566	**MAF[a]**	0.24/0.20	0.23/0.21	0.23/0.21	0.25/0.20	0.17/0.22	0.14/0.22	0.15/0.22
		**OR (95% CI)**	1.20 (0.76, 1.90)	1.09 (0.57, 2.10)	1.06 (0.62, 1.81)	1.28 (0.79, 2.08)	0.81 (0.33, 2.00)	1.84 (0.84, 4.04)	0.73 (0.36, 1.47)
		**p-value**	0.4	0.8	0.8	0.3	0.6	0.1	0.4
PTGS2	rs10911902	**MAF[a]**	0.17/0.14	0.16/0.14	0.10/0.15	0.10/0.16	0.28/0.14	0.05/0.16	0.22/0.13
		**OR (95% CI)**	1.36 (0.82, 2.28)	1.17 (0.56, 2.43)	0.67 (0.33, 1.38)	0.60 (0.31, 1.16)	2.08 (0.98, 4.38)	3.12 (0.96, 10.14)	1.57 (0.85, 2.91)
		**p-value**	0.2	0.7	0.3	0.1	0.1	0.1	0.2
PTGS2	rs5277	**MAF[a]**	0.11/0.11	0.14/0.11	0.09/0.12	0.10/0.11	0.08/0.11	0.17/0.10	0.10/0.11
		**OR (95% CI)**	1.08 (0.58, 2.01)	1.28 (0.58, 2.83)	0.78 (0.36, 1.69)	0.81 (0.41, 1.63)	0.66 (0.19, 2.26)	0.58 (0.27, 1.22)	0.81 (0.33, 1.97)
		**p-value**	0.8	0.5	0.5	0.6	0.5	0.1	0.6
SULT1A1	rs1968752	**MAF[a]**	0.35/0.40	0.29/0.40	0.47/0.38	0.39/0.39	0.50/0.38	0.40/0.39	0.39/0.39
		**OR (95% CI)**	0.79 (0.53, 1.19)	0.61 (0.33, 1.13)	1.47 (0.93, 2.33)	1.05 (0.69, 1.61)	1.58 (0.80, 3.13)	1.00 (0.57, 1.77)	0.92 (0.55, 1.56)
		**p-value**	0.3	0.1	0.1	0.8	0.2	1.0	0.8
TP53	rs1614984	**MAF[a]**	0.36/0.45	0.52/0.42	0.34/0.44	0.44/0.43	0.53/0.42	0.47/0.42	0.43/0.43
		**OR (95% CI)**	0.71 (0.47, 1.08)	1.54 (0.87, 2.72)	0.67 (0.41, 1.09)	1.05 (0.68, 1.62)	1.49 (0.73, 3.04)	0.60 (0.34, 1.06)	0.95 (0.56, 1.64)
		**p-value**	0.1	0.1	0.1	0.8	0.3	0.1	0.9
XPA	rs1800975	**MAF[a]**	0.33/0.35	0.45/0.33	0.28/0.36	0.35/0.35	0.36/0.34	0.36/0.34	0.35/0.35
		**OR (95% CI)**	0.96 (0.64, 1.44)	1.50 (0.87, 2.59)	0.72 (0.44, 1.17)	0.98 (0.64, 1.51)	1.03 (0.51, 2.07)	0.93 (0.54, 1.62)	1.03 (0.61, 1.75)
		**p-value**	0.8	0.1	0.2	0.9	0.9	0.8	0.9

		**MAF[a]**	0.20/0.22	0.29/0.21	0.22/0.22	0.21/0.22	0.22/0.22	0.28/0.21	0.17/0.22
XPA	rs3176757	**OR (95% CI)**	0.86 (0.54, 1.39)	1.47 (0.80, 2.72)	1.03 (0.60, 1.75)	0.91 (0.55, 1.52)	1.03 (0.46, 2.32)	0.71 (0.39, 1.31)	0.71 (0.37, 1.38)
		**p-value**	0.5	0.2	0.9	0.7	0.9	0.3	0.3
		**MAF[a]**	0.19/0.18	0.16/0.19	0.10/0.20	0.16/0.19	0.33/0.17	0.21/0.18	0.22/0.18
XPC	rs10468	**OR (95% CI)**	1.13 (0.68, 1.89)	0.83 (0.39, 1.79)	0.47 (0.22, 0.99)	0.84 (0.48, 1.48)	2.15 (1.02, 4.53)	0.87 (0.43, 1.76)	1.19 (0.64, 2.20)
		**p-value**	0.6	0.6	0.0	0.5	0.0	0.7	0.6
		**MAF[a]**	0.20/0.19	0.21/0.19	0.14/0.20	0.20/0.19	0.31/0.18	0.16/0.20	0.18/0.19
XPC	rs1106087	**OR (95% CI)**	1.18 (0.72, 1.94)	1.12 (0.56, 2.25)	0.68 (0.36, 1.31)	1.06 (0.62, 1.81)	1.79 (0.85, 3.77)	1.51 (0.70, 3.27)	0.89 (0.46, 1.70)
		**p-value**	0.5	0.7	0.3	0.8	0.1	0.3	0.7
		**MAF[a]**	0.20/0.19	0.21/0.19	0.14/0.20	0.20/0.19	0.31/0.19	0.16/0.20	0.18/0.20
XPC	rs2228000	**OR (95% CI)**	1.17 (0.71, 1.92)	1.11 (0.55, 2.22)	0.67 (0.35, 1.29)	1.05 (0.61, 1.79)	1.77 (0.84, 3.75)	1.55 (0.71, 3.36)	0.88 (0.46, 1.69)
		**p-value**	0.5	0.8	0.2	0.9	0.1	0.3	0.7
		**MAF[a]**	0.41/0.39	0.46/0.39	0.44/0.39	0.33/0.41	0.47/0.39	0.31/0.41	0.42/0.40
XPC	rs2228001	**OR (95% CI)**	1.07 (0.72, 1.58)	1.38 (0.80, 2.38)	1.28 (0.82, 2.01)	0.71 (0.46, 1.09)	1.30 (0.67, 2.53)	1.49 (0.83, 2.66)	0.98 (0.59, 1.62)
		**p-value**	0.7	0.3	0.3	0.1	0.4	0.2	0.9
		**MAF[a]**	0.20/0.16	0.14/0.18	0.11/0.19	0.17/0.17	0.31/0.16	0.16/0.18	0.17/0.17
XPC	rs2470458	**OR (95% CI)**	1.32 (0.82, 2.14)	0.77 (0.36, 1.67)	0.58 (0.29, 1.16)	0.99 (0.58, 1.68)	2.03 (0.97, 4.22)	1.23 (0.58, 2.60)	0.91 (0.47, 1.76)
		**p-value**	0.2	0.5	0.1	1.0	0.1	0.6	0.8
		**MAF[a]**	0.46/0.49	0.41/0.49	0.40/0.50	0.54/0.47	0.47/0.48	0.43/0.47	0.50/0.48
XPC	rs2607737	**OR (95% CI)**	0.91 (0.61, 1.35)	0.72 (0.41, 1.27)	0.68 (0.43, 1.08)	1.38 (0.90, 2.11)	0.93 (0.46, 1.89)	0.67 (0.38, 1.18)	1.12 (0.67, 1.87)
		**p-value**	0.6	0.3	0.1	0.1	0.8	0.2	0.7
		**MAF[a]**	0.40/0.39	0.46/0.39	0.44/0.38	0.32/0.41	0.47/0.39	0.31/0.40	0.42/0.39
XPC	rs2607772	**OR (95% CI)**	1.05 (0.71, 1.55)	1.40 (0.81, 2.42)	1.31 (0.84, 2.06)	0.69 (0.45, 1.07)	1.31 (0.67, 2.54)	1.46 (0.82, 2.61)	0.98 (0.59, 1.63)
		**p-value**	0.8	0.2	0.2	0.1	0.4	0.2	0.9
		**MAF[a]**	0.40/0.44	0.36/0.44	0.33/0.45	0.49/0.41	0.42/0.43	0.45/0.42	0.46/0.43
XPC	rs2733534	**OR (95% CI)**	0.90 (0.60, 1.37)	0.67 (0.37, 1.23)	0.63 (0.38, 1.03)	1.44 (0.92, 2.25)	0.84 (0.41, 1.70)	0.59 (0.33, 1.06)	1.11 (0.65, 1.88)
		**p-value**	0.6	0.2	0.1	0.1	0.6	0.1	0.7
		**MAF[a]**	0.30/0.26	0.27/0.27	0.20/0.28	0.27/0.27	0.39/0.26	0.26/0.27	0.25/0.27
XPC	rs2733537	**OR (95% CI)**	1.25 (0.80, 1.96)	0.94 (0.49, 1.80)	0.62 (0.35, 1.11)	1.00 (0.62, 1.62)	1.81 (0.87, 3.75)	1.17 (0.60, 2.27)	0.91 (0.50, 1.65)
		**p-value**	0.3	0.9	0.1	1.0	0.1	0.6	0.7
		**MAF[a]**	0.09/0.09	0.13/0.09	0.06/0.10	0.09/0.09	0.11/0.09	0.10/0.09	0.07/0.09
XPC	rs2733587	**OR (95% CI)**	1.05 (0.52, 2.13)	1.50 (0.61, 3.69)	0.53 (0.20, 1.42)	0.98 (0.46, 2.08)	1.29 (0.42, 3.97)	0.87 (0.33, 2.25)	0.78 (0.29, 2.10)
		**p-value**	0.9	0.4	0.2	1.0	0.7	0.8	0.6
		**MAF[a]**	0.21/0.26	0.20/0.25	0.23/0.25	0.33/0.23	0.08/0.26	0.34/0.24	0.24/0.25
XPC	rs3731149	**OR (95% CI)**	0.79 (0.48, 1.28)	0.68 (0.33, 1.40)	0.92 (0.53, 1.60)	1.80 (1.11, 2.91)	0.24 (0.07, 0.80)	0.59 (0.32, 1.09)	0.97 (0.53, 1.78)
		**p-value**	0.3	0.3	0.8	0.0	0.0	0.1	0.9
