# Supplementary material for: Gastrointestinal Stromal Tumors, Somatic Mutations and Candidate Genetic Risk Variants
Source: PLoS One. 2013 Apr 18;8(4):e62119. doi: 10.1371/journal.pone.0062119 (PMC3630216; doi:10.1371/journal.pone.0062119)
Supplement: Table S3 — P-values for sequence kernel association test (SKAT) for remaining genes, by mutation type. (PDF) [file pone.0062119.s003.pdf]

| Supplementary Table S3: P-values for sequence kernel association test (SKAT) for remaining genes, by mutation type | | | | | | | |
|---|---|---|---|---|---|---|---|
| **Gene** | **KIT exon 11 codon 557-8 deletion** | **KIT exon 11 insertion** | **KIT exon 11 other deletion** | **KIT exon 11 point mutation** | **Other KIT mutation** | **PDGFRA mutation** | **Wild type** |
| ADH1A | 0.8 | 1.0 | 0.1 | 0.6 | 0.1 | 0.1 | 0.3 |
| ADH1B | 0.1 | 0.6 | 0.4 | 0.9 | 0.5 | 0.2 | 0.3 |
| ADH1C | 0.1 | 0.3 | 0.4 | 0.5 | 0.2 | 0.5 | 0.2 |
| AHR | 0.8 | 0.7 | 0.8 | 0.1 | 0.5 | 0.5 | 0.3 |
| ALDH18A1 | 0.6 | 0.8 | 0.1 | 0.4 | 0.9 | 0.3 | 0.9 |
| ALDH1A1 | 0.5 | 0.7 | 1.0 | 0.4 | 0.2 | 1.0 | 1.0 |
| ALDH1A2 | 0.3 | 0.7 | 0.6 | 0.4 | 0.9 | 0.5 | 0.5 |
| ALDH1A3 | 0.6 | 0.3 | 0.2 | 0.8 | 0.4 | 0.8 | 0.7 |
| ALDH1B1 | 0.6 | 0.5 | 0.4 | 0.7 | 0.8 | 0.7 | 1.0 |
| ALDH1L1 | 0.4 | 0.2 | 0.3 | 0.8 | 1.0 | 0.4 | 0.1 |
| ALDH1L2 | 0.5 | 0.2 | 0.2 | 0.9 | 0.6 | 0.9 | 0.01 |
| ALDH2 | 0.5 | 0.03 | 0.9 | 0.6 | 0.7 | 0.2 | 0.2 |
| CYP1A2 | 0.1 | 0.3 | 0.6 | 0.5 | 0.9 | 0.3 | 0.9 |
| CYP2B6 | 0.4 | 0.2 | 1.0 | 0.1 | 0.9 | 0.5 | 0.1 |
| CYP2C8 | 0.4 | 0.4 | 0.1 | 0.7 | 0.8 | 0.9 | 0.2 |
| CYP2C9 | 0.5 | 0.9 | 0.3 | 0.3 | 0.6 | 0.4 | 0.1 |
| CYP2D6 | 0.7 | 0.3 | 0.02 | 0.3 | 1.0 | 0.8 | 0.2 |
| CYP2E1 | 0.5 | 0.5 | 0.9 | 0.9 | 0.04 | 0.3 | 0.7 |
| CYP3A4 | 0.2 | 0.4 | 0.4 | 0.7 | 0.3 | 0.9 | 0.6 |
| ERCC5 | 0.1 | 0.9 | 0.7 | 1.0 | 0.7 | 0.7 | 0.9 |
| G6PC | 0.6 | 0.1 | 0.5 | 0.2 | 0.5 | 0.4 | 0.4 |
| GSTP1 | 0.5 | 0.5 | 0.9 | 0.3 | 0.4 | 0.3 | 0.9 |
| GSTT1 | 0.2 | 0.6 | 0.02 | 0.1 | 0.7 | 0.04 | 0.8 |
| HIF1A | 0.4 | 0.7 | 0.3 | 0.5 | 0.8 | 0.2 | 0.8 |
| HNF4A | 0.6 | 1.0 | 0.4 | 0.1 | 0.3 | 0.6 | 0.7 |
| MDM2 | 0.2 | 0.7 | 0.2 | 0.7 | 0.3 | 0.6 | 0.4 |
| NAT2 | 0.4 | 1.0 | 0.3 | 0.6 | 0.2 | 0.3 | 0.1 |
| NFE2L2 | 0.8 | 0.7 | 0.1 | 0.4 | 0.3 | 0.7 | 0.5 |
| NOS2A | 0.3 | 0.5 | 0.2 | 0.2 | 0.3 | 0.2 | 0.7 |
| NQO1 | 0.9 | 0.5 | 0.03 | 0.5 | 0.8 | 0.5 | 0.1 |
| PTGS2 | 0.3 | 0.6 | 0.6 | 0.2 | 0.1 | 0.1 | 0.4 |
| SULT1A1 | 0.3 | 0.1 | 0.1 | 0.9 | 0.3 | 1.0 | 0.3 |
| TP53 | 0.1 | 0.2 | 0.2 | 0.5 | 0.2 | 0.1 | 1.0 |
| XPA | 0.8 | 0.2 | 0.3 | 1.0 | 0.9 | 0.6 | 0.7 |
| XPC | 0.6 | 0.6 | 0.1 | 0.3 | 0.1 | 0.2 | 1.0 |
